# Adsorptive removal of levofloxacin and antibiotic resistance genes from hospital wastewater by nano-zero-valent iron and nano-copper using kinetic studies and response surface methodology

**DOI:** 10.1186/s40643-022-00616-1

**Published:** 2023-01-09

**Authors:** Mohammed Taha Moustafa Hussien Hamad, Marwa E. El-Sesy

**Affiliations:** Central Laboratory for Environmental Quality Monitoring, National Water Research Center, Cairo, Egypt

**Keywords:** Levofloxacin, Antibiotic resistance genes, CuONPs, Nano-zero-valent iron, response surface methodology (RSM)

## Abstract

In the twenty-first century, water contamination with pharmaceutical residues is becoming a global phenomenon and a threat. Antibiotic residues and antibiotic resistance genes (ARGs) are recognized as new emerging water pollutants because they can negatively affect aquatic ecosystems and human health, thereby posing a complex environmental problem. These nano-adsorbents of the next generation can remove these pollutants at low concentrations. This study focuses on the chemical synthesis of copper oxide nanoparticles (CuONPs) and nano-zero-valent iron (nZVI) used as nano-adsorbents for levofloxacin removal from water samples and antibiotic-resistant genes. The CuONPs and nZVI are initially characterized by transmission electron microscopy, scanning electron microscopy, and X-ray diffraction. The levofloxacin adsorption isotherm on the CuONPS and nZVI shows the best fit with the Langmuir isotherm model, exhibiting correlation coefficients (*R*^2^) of 0.993 and 0.999, respectively. The adsorption activities of CuONPS and nZVI were fitted to a pseudo-second-order kinetic model with correlation coefficients (*R*^2^) of 0.983 and 0.994, respectively. The maximum levofloxacin removal capacity was observed at (89%), (84%), (89%), (88%) and (71.6) at pH 7 and adsorbent dose(0.06 mg/L), initial LEV concentration (1 mg/L), temperature 25 °C, and contact time 120 min for CuONPs. Removal efficiency was (91%), (90.6%), (91%), (89%), and (80%), at pH 7, adsorbent dose(0.06), initial LEV concentration (1 mg/L), temperature 35 °C, and contact time 120 min. The levofloxacin adsorption is an exothermic process for nZVI and CuONPs, according to thermodynamic analysis. A thermodynamic analysis indicated that each adsorption process is spontaneous. Several genera, including clinically pathogenic bacteria (e.g., *Acinetobacter_baumannii, Helicobacter_pylori*, *Escherichia_coli, Pseudomonas_aeruginosa, Clostridium_beijerinckii, Escherichia/Shigella_coli, Helicobacter_cetorum, Lactobacillus_gasseri, Bacillus_cereus, Deinococcus_radiodurans, Rhodobacter_sphaeroides, Propionibacterium_acnes, *and *Bacteroides_vulgatus*) were relatively abundant in hospital wastewater. Furthermore, 37 antibiotic resistance genes (ARGs) were quantified in hospital wastewater. The results demonstrated that 95.01% of nZVI and 91.4% of CuONPs are effective adsorbents for removing antibiotic-resistant bacteria from hospital effluent. The synthesized nZVI and CuONPs have excellent reusability and can be considered cost effective and eco-friendly adsorbents.

**Graphical Abstract:**

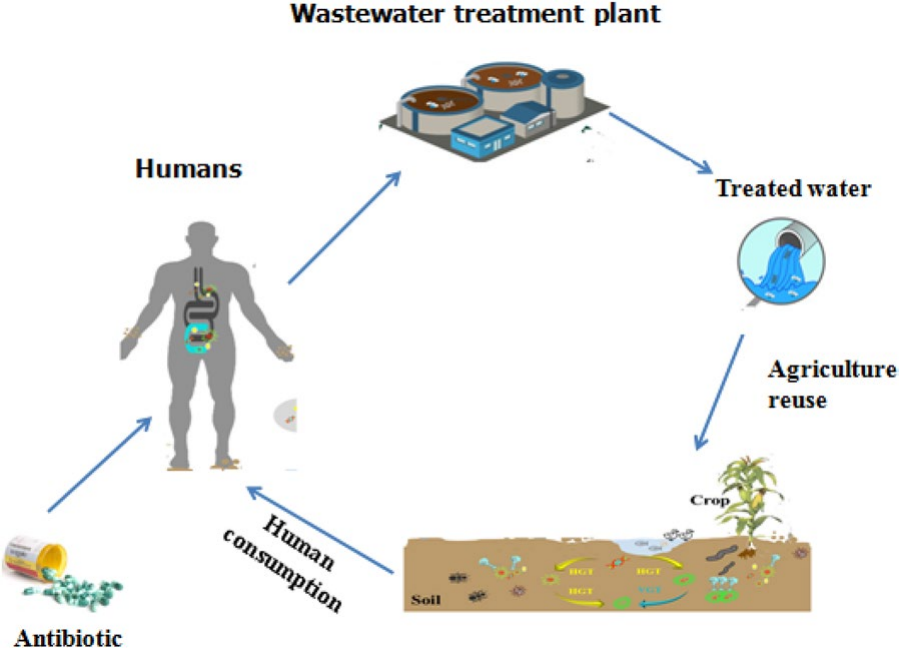

## Introduction

Water scarcity is increasing globally, especially in arid and semiarid regions such as, South Asia, Southern Africa, and the Middle East (Gatica and Cytryn 2013). The pollution of water resources by pharmaceutical waste is one of the greatest current challenges and most serious environmental problems facing the community, particularly in arid and semiarid regions where the demand for water is increasing daily due to the population?s exponential growth and rising living standards (Alexandre-franco and Fern 2020; Tan and Shuai 2015) the pharmaceuticals and drugs in water pose serious hazards to public health and aquatic ecosystems. These pollutants have the potential to disrupt the biological balance and photosynthetic cycle of plants as well as the enzymatic, hormonal, and genetic systems of humans (Patel et al. 2019). Therefore, it is essential to control these pollutants emitted from wastewater treatments in water bodies, before reaching soil and water, as well as their removal from the environmental matrix, particularly for human-consumed water (Khajuria and Kaur 2017). Antibiotics are a component of pharmaceutical chemicals. They are extensively used in treating various bacterial infectious diseases in humans, animals, poultry, and fish. The effects of excessive chemical usage can reach aquatic bodies, including surface and ground water, through various human-induced activities like waste streams from hospitals and the pharmaceutical and veterinary industries (from manufacture to disposal) (Koch et al. 2021). The annual antibiotic consumption has been estimated at 162,000 ton for China, 13,000 ton for the United States, and between 5000 and 10,000 ton for European countries (Alnajrani and Alsager 2020). Antibiotic resistance has emerged as a serious global health problem. Scientists estimate up to 10 million deaths per year by 2050 if it is not successfully combatted (Li et al. 2018). Antibiotics, such as sulfonamide, fluoroquinolone, and macrolide, have different half-lives in the environment, and some are highly persistent (Ziembi?ska-Buczy?ska et al. 2015). Consequently, their contamination levels in the environment have been increasing. The half-life values of levofloxacin, norfloxacin, erythromycin, and ofloxacin surface water are 6.3,77,17, and 10 days, respectively (Ziembi?ska-Buczy?ska et al. 2015). Ciprofloxacin, trimethoprim, clarithromycin, erythromycin, azithromycin, norfloxacin, and ofloxacin are among those frequently found in conventional water and wastewater treatment effluents. Hospitals account for the consumption of large quantities of pharmaceuticals every day. In most cases, hospital wastewaters consist of antibiotics, antibiotic-resistant bacteria, disinfectants, and detergents, which are directly dumped into the public wastewater system without any pre-treatment. This poses risk effects on the biological balance of natural media (Al-Gheethi et al. 2015; Perrodin et al. 2016). In hospitals, large quantities of the pharmaceuticals are consumed daily. In most cases, hospital of wastewater containing of antibiotics, antibiotic-resistant bacteria, disinfectants, and detergents are dumped directly into the public wastewater system without any pre-treatment, posing a threat to the biological equilibrium of natural media (Al-Gheethi et al. 2015; Perrodin et al. 2016). Four antibiotics, namely chlortetracycline, tylosin, sulfamethazine, and virginiamycin, have been detected in plant tissues with?<?10 µg kg^?1^ concentrations. According to recent studies, more than 250 antibiotics can be found in the wastewater of pharmaceutical manufacturers (Rodriguez-Mozaz et al. 2020). Antibiotics are frequently detected in sludge (Sabri et al. 2020), surface water (Anh et al. 2021), underground water (Szekeres et al. 2017), drinking water (Li et al. 2018; Yao et al. 2021) and sediments (Al-Khazrajy and Boxall 2016). Fluoroquinolones and B-lactams are two chemical classes that are widely used to treat infectious diseases throughout the world. Fluoroquinolones are antibiotics used to treat illnesses caused by Gram-negative bacteria like Enterobacteriaceae. Ciprofloxacin, gemifloxacin, levofloxacin, moxifloxacin, norfloxacin, and ofloxacin are examples of fluoroquinolones (Ezelarab et al. 2018). Levofloxacin is utilized to treat various human and animal diseases. It continues to appear in the aquatic environment because of its insufficient metabolization in human and animal bodies (Felis et al. 2020). Sulfamethoxazole and trimethoprim have been reported to range from 144 to 731 and 40 to 43 ng/L, respectively, in wastewater treated in Saudi Arabia (Al Qarni et al. 2016). In China, 20 and 17 antibiotics have been detected in influent and effluent samples, with erythromycin, ofloxacin, sulfamethoxazole, and norfloxacin being the most frequently detected ones (Faleye et al. 2018). In Egypt, antibiotic residues have been found in wastewater, with 99.04, 70.06, and 119.24 mg/L concentrations for amoxicillin, ampicillin, and dicloxacillin, respectively. In the Sohag Wastewater Treatment Plant in Egypt, 12 antibiotics have been detected in raw wastewater, treated effluent, and agricultural drainage water at concentrations ranging from 109 to 469 ng/L. In Pakistan, the levels of ciprofloxacin, levofloxacin, and ofloxacin were reported as 331.15, 6.63 and 2.54 mg L^?1^, respectively, in wastewater streams of Rawalpindi/Islamabad (Altaf et al. 2021). The development of antibiotic resistance genes (ARGs) is linked to the release of residual antibiotics into the aquatic environment. The development of antibiotic resistance genes (ARGs) has been gaining attention as an emerging environmental pollutant, that poses a harm threat to human health. The effluent from wastewater treatment plants (WWTPs) is recognized as a source of ARGs released into the environment, even after chlorine or UV light disinfection (Li et al. 2017). Thus, the discharge of WWTP effluents may facilitate the spread of antibiotic resistance in receiving habitats, such as rivers and wastewater-irrigated soils (Zhang et al. 2016). Horizontal gene transfer (HGT) can transfer ARGs to bacteria of the same or different species. ARGs can persist in the environment even after selection pressures have (Jutkina et al. 2016). Soil, surface water and drinking water contain antibiotic-resistant bacteria (ARBs) and antibiotic resistance genes (ARGs) (Ben et al. 2009). According to the World Health Organization (WHO), antibiotic resistance is one of the three greatest threats to public health in the twenty-first century (Dhingra et al. 2020). In a recent report, the Inter-Agency Coordination Group on Antimicrobial Resistance of the United Nations estimated that at least 700,000 individuals each year pass away people die annually (Felis et al. 2020). Some environmental bacteria, such as *Arcobacter*, *Aeromonas*, *Corynebacterium*, *Clostridium, Nitrosomonas* and *Clostridium* were identified as potential hosts for ARGs that contribute to the spread of antibiotic resistance in activated sludge, influent, and effluent samples in form the United States and China (Tong et al. 2019). High frequencies of 15 tetracycline resistance genes (e.g., tet (A,B,C, tet D,E, G,K, L,M,O) and four sulfonamide resistance genes (sul1,2,3, and sulA) have been detected in wastewater treatment plants (WWTPs), river water, soil, and aquaculture farms (Chen and Zhang 2013). Antibiotic residues may be detectable in receiving waters, as indicated by the finding of number of researchers who have found that conventional wastewater treatment methods are incapable of removing them entirely. Physical and chemical (Khajuria and Kaur 2017). Numerous techniques for antibiotic removal from water have been widely studied. These include biodegradation (Al-Gheethi et al. 2015), electron pulse radiolysis (Wang et al. 2020), coagulation (Alnajrani and Alsager 2020), ozonation (Becker et al. 2016), electrochemical advanced oxidation, photocatalysis, and photoelectron catalysis (Akbari et al. 2021; Vaiano et al. 2015). However, these approaches cannot achieve high efficiencies because the relatively high cost limits their large-scale application and time consumption. Moreover, they are largely ineffective and end up adding more pollutants into the environment (Magesh et al. 2021). Over the years, studies have mainly focused on new technologies, such as new-generation adsorbents (nano-adsorbents), for wastewater treatment and water supply needed to eliminate these pollutants (Basheer 2018). Nano-adsorbents are a new generation of adsorbents that scientists are interested because they have a high of adsorption, capacity, a surface area and a large number of active sites for interacting with various pollutants and can remove any trace pollutants (Dalal et al. 2019). Research states that since the beginning of the twentieth century, it has been the most widely used, technically and biologically significant substance, with practical applications in industrial and environmental protection (Aguilar-Pérez et al. 2021). The adsorption-based method is a promising technology for antibiotic removal because of its advantages of cost effectiveness, environmental friendliness, and wide flexibility range (Arica et al. 2022; Liu et al. 2017). Its performance is greatly dependent on the adsorbent activity (Abegunde et al. 2020). Various adsorption-based substances, including hydrous manganese oxide, aluminum, cobalt ferrite, gold, maghemite, copper oxide clay minerals, iron hydrous oxides, synthetic polymers terpolymer resin, and activated carbon, are already being used to remove antibiotics (Bayramoglu et al. 2020; Rahmani et al. 2017; Shi et al. 2017; Sousa et al. 2018). This study sought to: (1) determine the occurrence of antibiotics in the drain and effluent water of hospitals wastewater treatment plants; (2) evaluate the efficiency of nano-zero-valent and nano-copper as an adsorbent in the adsorption of levofloxacin from aqueous solutions. The using adsorption mechanism proposed according to the results of the effect of operating variables, the adsorption kinetics, the adsorption isotherm and response surface methodology and, determine the abundance profiles of ARGs in raw and treated hospital wastewater using metagenomic analysis.

## Materials and methods

### Chemicals and reagents

#### Chemicals

**Fig. 1:**
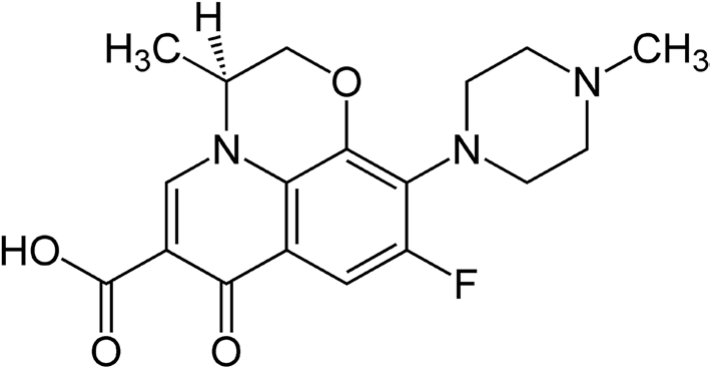
Chemical structures of levofloxacin

Throughout this work, analytical-grade chemicals were Fe(III) chloride and copper(II) sulfate pentahydrate salt, CuOSO_4_.5H_2_O, with a 99.8% rating, as well as sulfuric acid, sodium hydroxide and methanol, extracted from Fe(III) chloride (Merck). Powder with a 97% purity level of levofloxacin (Fig. 1) was obtained from Sigma-Aldrich, Egypt. The various concentrations of the solutions were prepared using distilled, deionized water.

### Sample collection and processing

Bilbeis drain water samples were collected, and the Bilbeis hospital wastewater treatment plant (BS-WWTP) is located in the Egyptian governorate of Ash Sharqia. All samples were collected in sterile polyethylene bottles, for DNA extraction while 1 L of water was stored in brown glass bottles for antibiotic analysis.

### Quantification of antibiotic concentrations in samples

The antibiotics were analyzed using high-performance liquid chromatography (HPLC), a Waters 2690 and 996 Alliance HPLC system outfitted with a photodiode array detector was utilized for the analysis (Suke et al. 2015).

### Nanomaterials

#### Preparation of copper oxide nanoparticles

Copper oxide (CuO) nano-powder was manufactured using the sol?gel technique. In a thoroughly cleaned round-bottom flask, an aqueous solution of copper (II) sulfate pentahydrate, CuOSO_4_·5H_2_O, was prepared using (0.2 M). The aforementioned aqueous solution was continuously as mixed while 1 mL of glacial acetic acid was added and heated to 100 °C. When the pH reached 7, 8 M NaOH was added gradually to the hot solution while stirring vigorously. A large amount of black precipitate began to for immediately and was air dried for 24 h (Dessouki et al. 2014).

#### Preparation of zero-valent iron nanoparticles

Subsequently, for the synthesis of nano-zero-valent iron, 0.5409 g of FeCl_3_·6H_2_O were dissolved in a solution of ethanol and water (24 mL methanol plus 6 mL deionized water), and the mixture was vigorously stirred. Excess borohydride is required for better iron nanoparticle growth. Hence, 0.1 M solution was made by dissolving 0.3783 g sodium borohydride in 100 mL of deionized water. The black iron nanoparticles were separated from the liquid phase by vacuum filtration. Finally, the nanoparticles were dried overnight at 50 °C in an oven (Sudhanya and Chinnamma 2018).

### Adsorbent characterization

The crystalline phases of the zero-valent iron and the copper oxide nanoparticles were characterized through X-ray diffraction (XRD) using a 6100 series X-ray diffractometer (Shimadzu Instrument) with Cu?k? radiation with 1.54? wavelength. The nZVI and CuONP surfaces were examined using a JEOL JEM-2100 transmission electron microscope. Subsequently, using scanning electron microscopy (SEM), the morphological structure. The surfaces of nanoZVI and CuONPs were analyzed with a JEOL JEM-2100 transmission electron microscope (TEM).

### Batch of levofloxacin adsorption by nZVI/CuONPs

An adequate amount of levofloxacin samples (25.0 mg) was dissolved in distilled water in a 1000-mL volumetric flask to produce a stock solution with 1000 mg/L concentration. Further 10?50 mg/L concentrations were also prepared from the stock by dilution. Adsorption was conducted through the batch technique using 0.01 g adsorbent with 10 mL of 50 mg/L levofloxacin solution. The mixture was shaken at 200 rpm for 6 min and left for contact times of 10, 15, 30, 45, 60, 90, and 120 min at 25 °C. The samples were then centrifuged for 20 min at 4000 rpm to separate the supernatant from the adsorbents. In the adsorption studies, different adsorbent dosages (i.e., 0.01, 0.02, 0.04, 0.05, and 0.06 g/L), contact times (i.e., 10?120 min), pH (i.e., 3, 5, 7, 9, and 10), temperature ranges (i.e., 298, 308, 318, and 328 K), and initial levofloxacin concentrations (i.e., 1, 2, 4, 6, and 8 mgL^?1^) were employed. The pH of the levofloxacin solution was adjusted by adding HCl or NaOH 0.1 M until the desired pH was attained. The levofloxacin concentration in the supernatant solution was measured by the spectrophotometric method using the Orion Aquamat 8000 spectrophotometer at 287 nm wavelength(Mahmoud et al. 2020).The nZVI and CuONP particles were recycled numerous times until they lost activity. The experiments were performed at least thrice. Equations (1) and (2) were used to determine the levofloxacin removal % and the adsorption capacity of the adsorbents as follows:

1 $$Q=\frac{\left(Ci-Cf\right)}{m}\times V,$$

2 $$R=\frac{(Ci-Cf)}{Ci}\times 100,$$

where *Q*a nd *V* are the adsorption capacity of the adsorbent (mg/g) and the sample volume (L), respectively; *Ci* and *Cf* are the initial (mg/L) and residual (mg/L) levofloxacin concentrations in the solution, respectively, after the adsorption process; and *R* and *m* are the levofloxacin removal percentage and the adsorbent mass (g) in the adsorption process, respectively.

### Adsorption isotherms

The 1, 2, 4, 6, and 8 mg L^?1^ initial levofloxacin concentrations were used to process the adsorption isotherms. Accordingly, 10 mg nZVI and CuONPs were combined with 50 mL levofloxacin. The mixture was thoroughly shaken for 10 min and filtered. Finally, a UV?visible spectrophotometer was used to measure the levofloxacin concentration in the filtrate. Equation (2) was employed to calculate the levofloxacin percentage removal. The most common equations characterizing the data from the adsorption equilibrium are the Langmuir and Freundlich isotherms illustrated in Eqs. (3) and (4), respectively (Nasseh et al. 2019):

3 $$\frac{{{C}}_{{{e}}}}{{{{q}}}_{{{e}}}}=\boldsymbol{ }\frac{{{{C}}}_{{{e}}}}{{{{Q}}}_{{{m}}{\varvec{a}}{{x}}}}\boldsymbol{ }+\boldsymbol{ }\frac{1}{{{{Q}}}_{{{m}}{{a}}{{x}}}{{{K}}}_{{{L}}}},$$

4 $$ln{q}_{e}=ln{K}_{F}+\frac{1}{n}ln{C}_{e},$$

where *C*_*e*_ represents the equilibrium concentration of levofloxacin in the solution (in mg L^1^); *q*_*e*_ is the equilibrium amount of levofloxacin adsorbed by the nZVI and CuONPs (in mg g^?1^); and *Q*_*max*_ and b are the maximum adsorption capacity (in mg g^?1^) and the Langmuir constant, respectively. These parameters were calculated from the plot of *C*_*e*_/*q*_*e*_ vs. *C*_*e*_. The relationship showed a straight line with parameters 1/*Q*_*max*_ for the slope and 1/*Q*_*max*_*b* for the intercept. *K*_*F*_ and *n* were the Freundlich parameters. A straight line with a slope of 1/*n* and an intercept of *lnK*_*F*_ was achieved when *lnq*_*e*_vs*lnC*_*e*_ was plotted. The adsorption effectiveness was revealed by the slope *n*, while the adsorption capacity was depicted by the *lnK*_*F*_ intercept (Kerkez-Kuyumcu and Salam 2016). The Temkin model is linearly expressed as follows Eq. (5):

5 $${q}_{e}=\frac{RT}{b}\mathit{ln}\left({K}_{T}\right)+\frac{RT}{b}ln\left({C}_{e}\right).$$

The Temkin constant (J/mol) is denoted by the *b*?=?*RT*/*b*1 expression. The universal gas constant, *R*, is 8.314 J/mol. The absolute temperature (°K) is *K*_*T*_. The equilibrium binding constant (L/g) and the adsorption heat (kJ/mol) are denoted by *kt* and *b*1, respectively. The magnitude of the *b*1 value indicated that levofloxacin was quickly removed at the beginning of the process. The smallness of the *kt* value suggested that the levofloxacin molecules were only weakly bonded to the composite.

### Thermodynamic study

The thermodynamic studies were completed, and their parameters were established according to the procedure presented here. Equations (6) and (7) were used to calculate the thermodynamic parameters, including the changes in the free energy (?*G*°), enthalpy (?*H*°), and entropy (?*S*°), using 50 mL mixture of 1 mg L^?1^ levofloxacin and 0.01 mg L^?1^nZVI and CuONPs shaken for 10 min at various temperatures of 298, 308, 318, and 328 K (Eq. 7):

6 $$\Delta G^\circ =-RTInkc,$$

7 $$\Delta G^\circ =\Delta H-T\Delta S^\circ .$$

?*H*° and ?*S*°were estimated from the plot of *lnkc* versus 1/*T*, where a straight line was formed. The enthalpy and entropy values were determined from the slope and the intercept, respectively.

### Adsorption kinetics

Several models can be used to investigate the adsorption process mechanisms and explanations based on experimental data. The pseudo-primary and secondary rate equations are the most applicable models (Fan et al. 2017). Eqs. (8) and (9) describe the pseudo-first-order and pseudo-A second-order adsorption rates developed by Abramian and El-Rassy (2009):

Pseudo-first-order equation:

8 $$\mathrm{log}\left({q}_{e}-{q}_{t}\right)=\mathrm{log}{q}_{e}-\left(\frac{{k}_{1}}{2.303}\right)\mathrm{t}$$

Pseudo-A second-order equation:

9 $$\frac{t}{{q}_{t}}= \frac{1}{{k}_{2}{q}_{e}^{2}}+ \frac{1}{{q}_{e}}\mathrm{t},$$

where *q*_*t*_ represents the adsorption capacity at a given time; *q*_*e*_ denotes the adsorption capacity under equilibrium conditions; and *k*_1_ and *k*_2_ denote the adsorption rate constants. According to Yi et al. (2016), *q*_*e*_ and *q*_*t*_ are the equilibrium and adsorption capacities (mg g^?1^), respectively, at time *t*; *k*_1_ is the pseudo-first-order constant (min^?1^); and *t* is the contact time. The rate constant (*k*_1_) was determined from the slope of the log(*q*_*e*_???*q*_*t*_) and *t* plots. The slope of plot*t* against *t*/*q*_*t*_ was used to compute *q*_*e*_. The second-order rate constant (*k*_2_) (g mg^?1^ min^?1^) was determined from the intercept (Alsager et al. 2018).

### Determination of zero-point charge

In order to determine the zero-point charge (pHpzc), a 0.1 M KCl solution was used.

In an Erlenmeyer flask 0.05 g of CuONPs and nZVI, along with 20 mL of pH 3 to12 KCl solution, were added and kept for 24 h. On a digital pH meter (Multi 9620 IDS-pH meter, WTW, Germany), the initial pH (pHi) of the solution was adjusted using HCL or NaOH. After 24 h, the solution's final pH (pHf) was determined. Adsorbent pHpzc was determined from the graphs of pHf?pHi versus pHi that were displayed (Parvin et al. 2021).

### Optimization of the levofloxacin removal from solution using CuONPs and nZVI

Subsequently, using the Design-expert-13 software?s Box?Behnken design method, antibiotics removal from aqueous matrices using CuONPs and nZVI was optimized. This experimental design included the parameters pH value, the levofloxacin concentration in solution, the dose adsorbent, and the contact time for the retention of antibiotics on CuONPs and nZVI. Minimum, medium and maximum working temperatures were set to 25 °C. Likewise, the pH was varied between 3 and 10, the antibiotic solution volume between 1 mg/L to 8 mg/L, the contact time 15 and 120 min, and the nanomaterial dosage between 0.01 to 0.04 mg/L. Table 1 displays the range and levels of the experimentally investigated Box?Behnken variables.

**Table 1:** Range of codes used in the Box?Behnken design for variables and their real experimental values

Code	Variables			
	pH	Initial concentration	Time (minute)	Dosage
-1	3	2	15	0.01
0	6	5	40	0.03
1	9	8	65	0.04

### DNA extraction and detection of resistance genes ARGs in water samples

Hospital wastewater samples were serially diluted in phosphate-buffered saline, and 100 ?L volumes of samples were spread-plated in triplicate and cultured on lysogeny broth agar at 30 °C for 48 h to determine the concentration of cultivable bacteria in the water sample. Subsequently, 100 ?L of sample water was filtered through a 0.2-?m pore size membrane filter (Sartorius, Germany). A NanoDrop spectrophotometer was used to determine the DNA's concentration and purity. When DNA extraction for molecular analysis was complete, the membrane was stored at ??20 °C (Le et al. 2018). Quantitative PCR was used to quantify the B-Lactam resistance genes blaOXA-1, blaTEM, blaOXA-10, blaTEM-1, blaDHA-1, blaSHV-1, and blaGES-1, as well as the eight quinolone resistance genes qnrA, qnrS, qnrB-1, qnrB-4, qnrB-5, and qepA. By analyzing of the 16S rRNA gene, the relative abundances of ARGs was determined. Each experiment utilized both positive and negative controls. Positive controls were created by cloning target DNA into plasmids at various dilutions (Yao et al. 2021).

## Results and discussion

### Characterization of CuONPs and nZVI

#### XRD

**Fig. 2:**
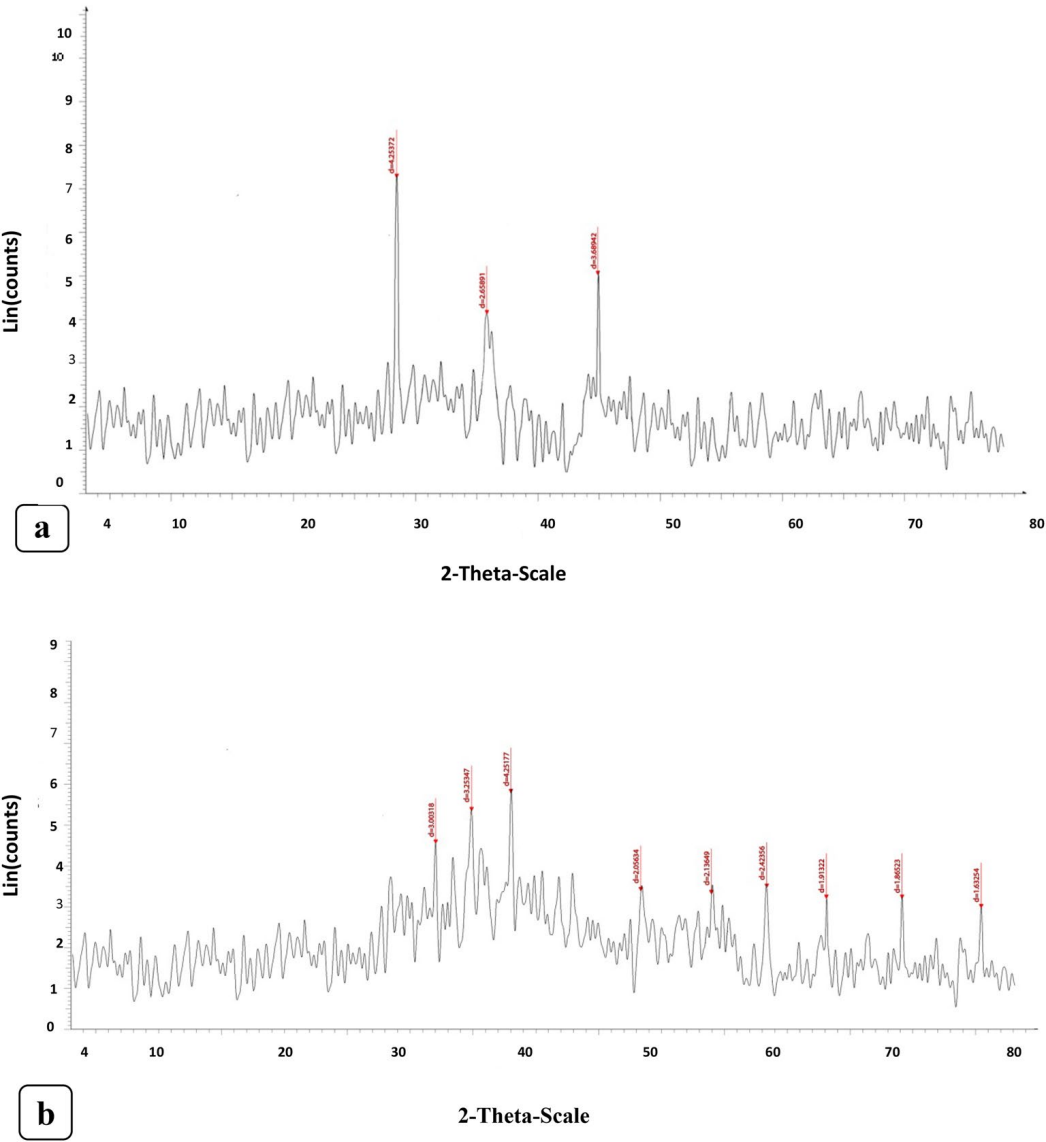
XRD patterns of synthesized: **a** nZVI and **b** CuONPs

The XRD diffractogram of the analysis demonstrated the crystalline character of the nZVI and CuONPs (Fig. 2a and b). The XRD spectrum displayed nine small and distinct diffraction peaks (i.e., 32.234, 35.543, 38.729, 48.809,54.263, 58.112, 63.331, 69.112, and 76.462) reflecting(110), (002), (111), (202), (020), (202), (113), (311),and (004), respectively, for the primitive structure of the CuONPs and three peaks at two theta angles at 29.125, 35.232, and 44.613 corresponding to the crystal planes of (210), (200), and (110), respectively, for the nZVI phase. According to the XRD analysis of the nZVI, the peak at 44.8 indicated the presence of pure metallic ?-Fe. Other peaks belonging to iron oxides and oxyhydroxides were also observed (Kumar et al. 2015). The XRD patterns of the nZVI nanoparticles and the monoclinic CuONPs were in a good agreement with JCPDS card nos. 06?0697 and 80?1916, respectively (El-shafei and Hefny 2018). Figure 2a and b shows the sharp peaks of the nZVI and the CuONPs, respectively, which indicate their crystallinities in nature. The Debye?Scherrer formula (*D*?=?*k*/cos) was used to calculate the average grain size of the CuONPs and nZVI synthesized during the bioreduction process. *D* and *k* indicate the average crystalline size (Å) and a 0:94 constant, respectively; *?* is the X-ray source wavelength (0.1541 nm); *?* is the angular line full width at half maximum intensity in radians; and *?* is Bragg?s angle (Chand and Trivedi 2019). The XRD pattern revealed the average crystallite sizes of the CuONPs and the nZVI as 22.6 and 24.05 nm, respectively.

#### Scanning electron microscopic (SEM) study

**Fig. 3:**
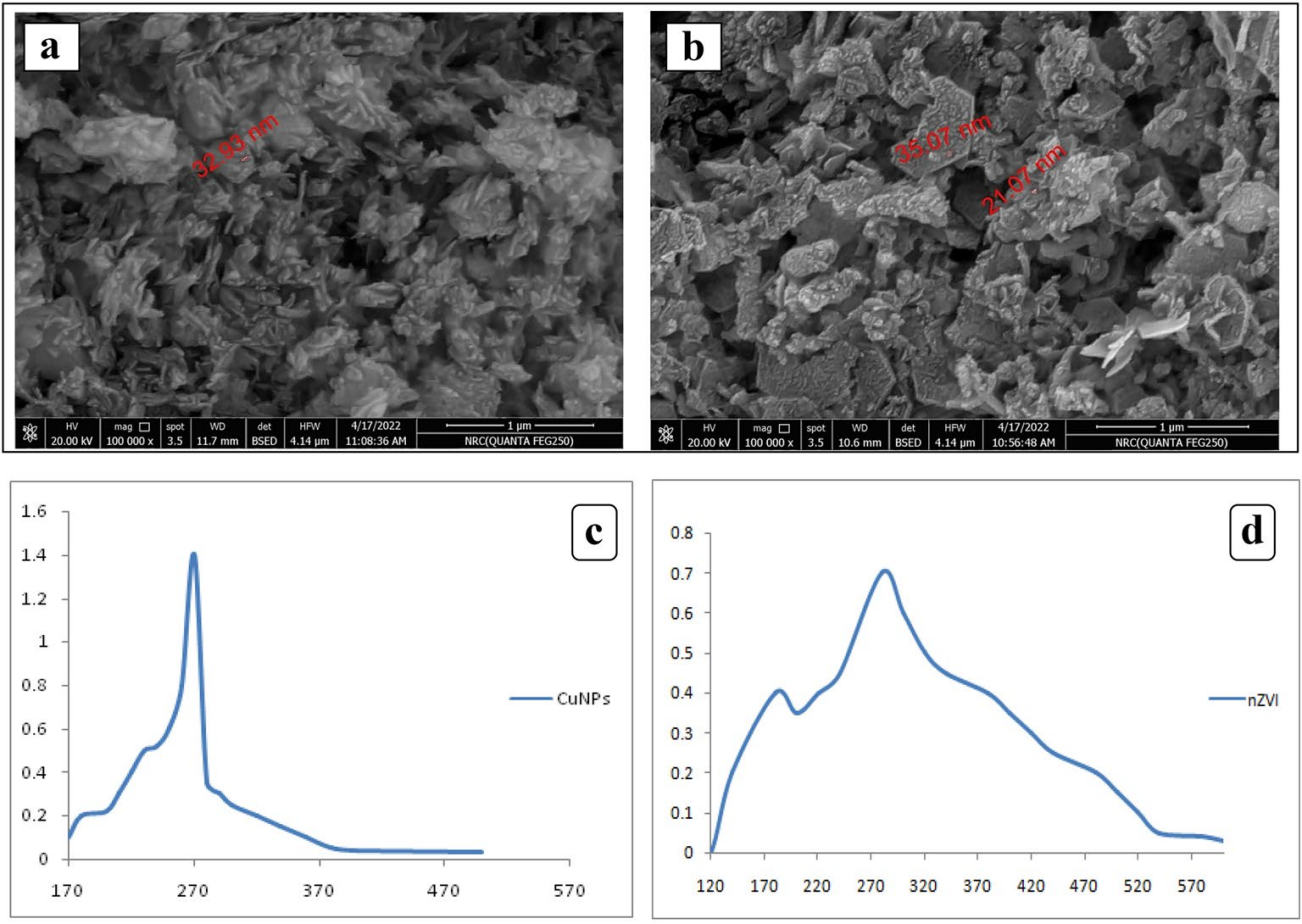
SEM images of the adsorbents of levofloxacin by **a** CuONPs and **b** nZVI, UV?visible spectroscopy of **c** CuONPs and **d** nZVI

Subsequently, the surface morphology of chemically synthesized CuONPs and nZVI was analyzed using a scanning electron microscope to determine their morphological structure. The surface properties of CuONPs and nZVI were evaluated after levofloxacin adsorption for the current study, as shown in (Fig. 3a and b): some spherical, polyhedral, and irregular particles with an average diameter between 21 and 35 nm for CuONPs and between 23 and 32 nm for nZVI. The surface morphology after adsorption confirms the action of thermal decomposition by displaying a uniformly thick, uniformly covered by the drug. The absorption spectrum of CuONPs and nZVI is shown at 270 nm and 280 nm, respectively, in Fig. 3c and d.

#### Characteristics of CuONPs and nZVI (TEM)

**Fig. 4:**
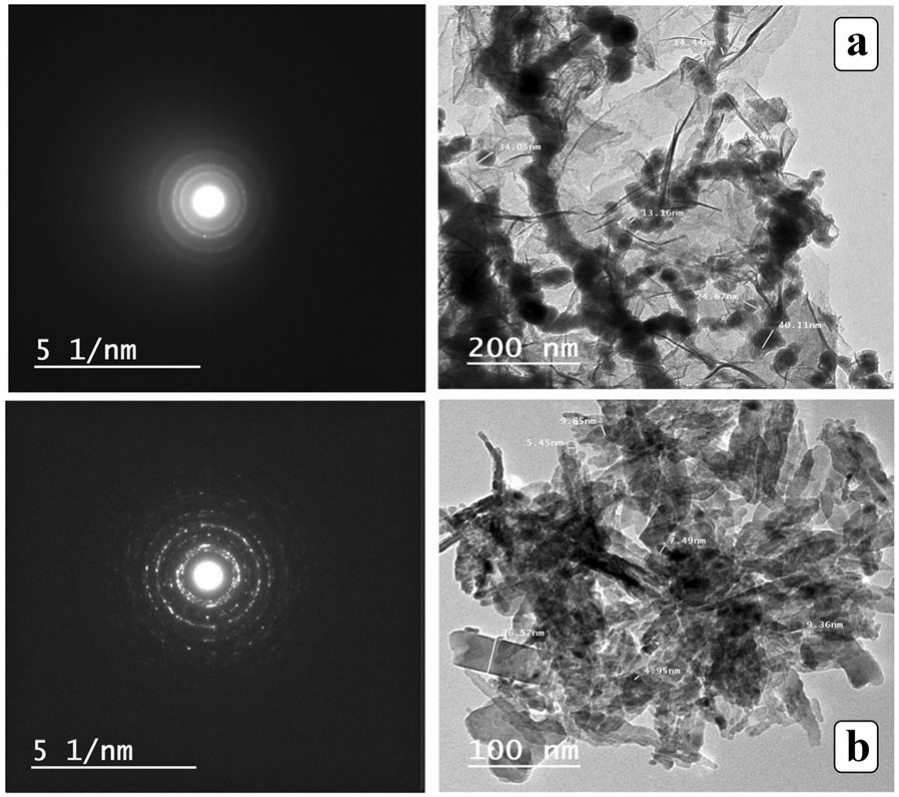
Transmission electron microscopy (TEM) images of a nZVI and b CuONPs

The TEM analysis yielded morphological, topographic, and crystallographic data. A high-resolution TEM image (Fig. 4a and b) revealed that the CuONPs and nZVI nanoparticles have an irregular shape, but are essentially spherical, with average diameters ranging from 3.59 to 37.7 nm and 5.48 to 36.75 nm for CuONPs and nZVI nanoparticles, respectively. Consequently, these particles fall within the range of 100 nm to 1 nm. In addition, there is a minimal aggregation of these nanoparticles, as their dispersion appears to be monodisperse.

#### CuONP and nZVI transmission electron microscopy (TEM) characteristics

In this work, the TEM analysis provided the morphological, topographical, and crystallographic information. The high-resolution TEM images in Fig. 4a and b illustrate the irregular forms of the CuONPs and the nZVI, although most are essentially spherical with average diameters 3.59 to 37.7 and 5.48 to 36.75 nm for the former and the latter, respectively. These particles fell within the 100 nm to nanometer range. A slight agglomeration was observed due to these nanoparticles being monodispersed in their dispersion.

#### Effect of pH on levofloxacin adsorption

**Fig. 5:**
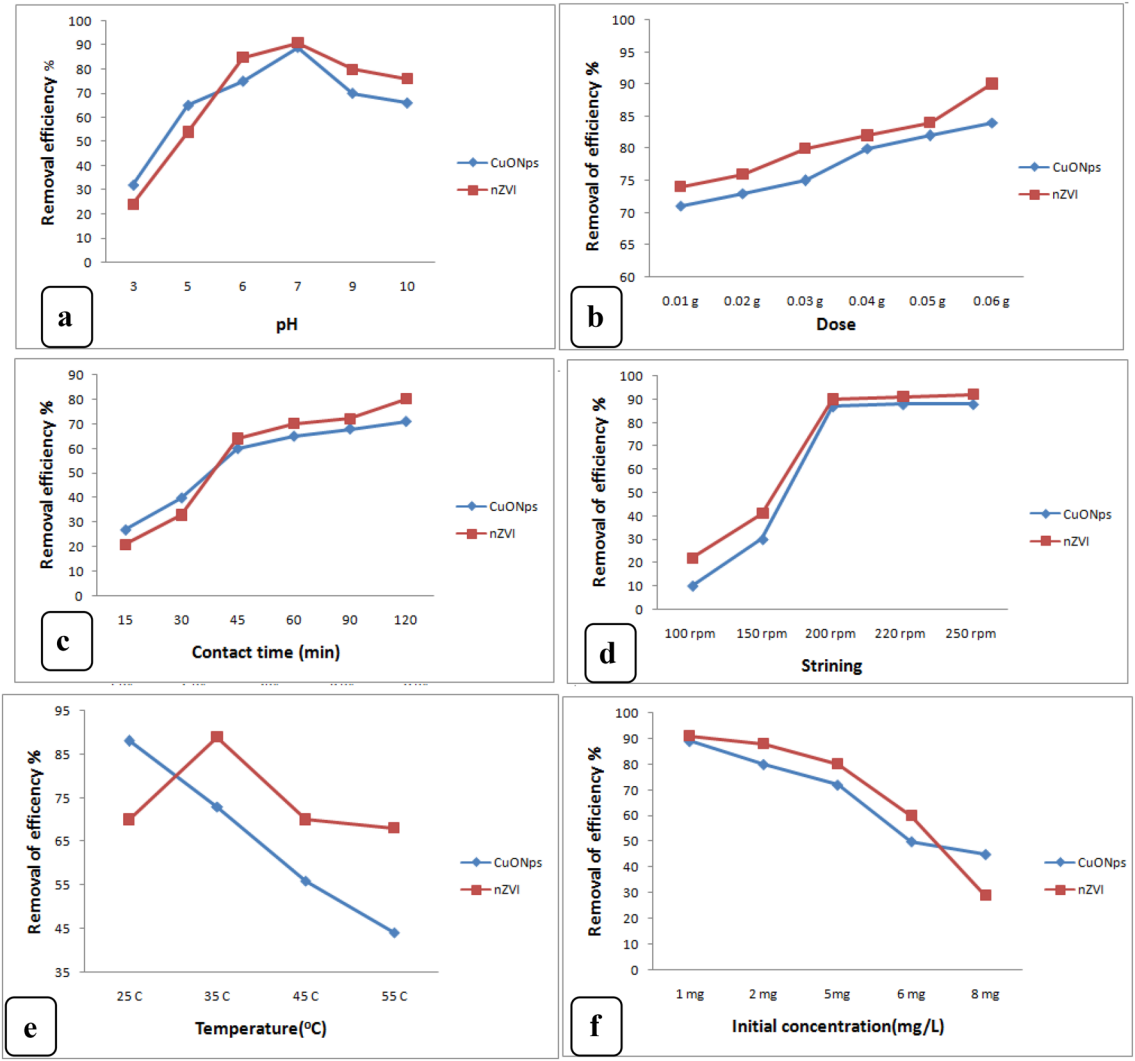
The influence of **a** pH (antibiotic conc. 1 mg/L, time 120 min, agitation 200 rpm, temp 308 K, dosage 0.01 g), **b** adsorbent dosage (pH 7.0, antibiotic conc. 1 mg/L, agitation 200 rpm, time 120 min, Temp 308 K, **c** contact time (pH7.0, dye conc. 0.01 mg/L, agitation 200 rpm, Temp 308 K, dosage 0.01 g), **d** stirring(antibiotic conc. 1 mg/L, time 120 min, temp 308 K, dosage 0.01 g), **e** temperature (antibiotic conc. 1 mg/L, time 120 min, agitation 200 rpm, dosage 0.01 g) and **f** antibiotic concentration (pH 7.0, time 120 min, Temp 308 K, agitation 200 rpm, dosage 0.01concentration) on levofloxacin uptake onto nZVI and CuONPs

The pH value is an essential element influencing the properties of levofloxacin and the adsorbent in the adsorption process. The adsorption percentage of levofloxacin clearly decreased with the pH increase from 3 to 6 (Fig. 5a). The levofloxacin solubility was even worse in the acid solution, which may possibly result in its low removal efficiency. The CuONP and nZVI surfaces gained a positive charge by absorbing H^+^ ions as a result of an increase in the H^+^ ion concentration of the system. Fig. 5a shows that the maximum levofloxacin removal capacities at pH 7 were 89 and 91% for the CuONPs and nZVI, respectively. However, a pH (7) increase resulted in more negatively charged sites. At the natural pH the levofloxacin present in the solution in zwitterionic form increases the electrostatic interaction between it and CuONP and nZVI, improving the adsorption. At high pH, the levofloxacin present undergoes repulsive interaction between OH^?^ anions. For levofloxacin, there is protonation of the amine group at a pH below 6.1 and deprotonation of the negatively charged carboxylic group. The maximum adsorption corresponded to the zwitterionic species (AbuKhadra et al. 2020). The adsorbate quantity extracted from a solution depends on the availability of the internal pore spaces present in the adsorbents used. An extremely potent electrostatic attraction formed between the antibiotic molecule and the negatively charged CuONP and nZVI surfaces because of the negative charge on these surfaces at high pH. This attraction resulted in the maximum levofloxacin adsorbed in the surface. The CuONPs and the nZVI had pHPZC values of 9.1 and 6.1, respectively. At a pH below the pHPZC, the surface became positively charged, and the H^+^ concentration was high. These factors competed with levofloxacin for available adsorption sites, resulting in a levofloxacin uptake reduction. The negatively charged adsorbent surface at pH?>?pHPZC favored the levofloxacin uptake because of the higher electrostatic force of attraction. The adsorbent surface showed the maximum amount of negative charge at pH 7. The surface charge intensity and the adsorption capacity did not increase when the pH was further increased. Accordingly, adsorption investigations were conducted at pH 7 (Davoodi et al. 2019). Mahmoud et al. (2020) detected that the removal percentage of LEVO was effective at pH 7 due to the decreasing electrostatic repulsion between the protonated amine groups on LEVO molecules and the positive charge on NBent-NTiO2-Chit surfaces.

#### Effect of n ZVI and CuONPs dosage on levofloxacin adsorption

The relationship between the CuONPand nZVI doses and the levofloxacin removal percentages was evaluated. The quantity of adsorbate to be removed from a solution was determined by the availability of the internal pore spaces in the adsorbents used. A lower adsorbent dosage is required to remove the adsorbate from the solution when the adsorbent has a greater pore space. A higher adsorbent dosage provides more pore spaces for adsorption. In this work, the optimum CuONP and nZVI dosage for achieving the highest levofloxacin removal rate was 0.06 mg (Fig. 5b).The levofloxacin adsorption rate for the nZVI sharply increased from 74 (0.01 mg nZVI dosage) to 82% (0.04 mg nZVI dosage). Its efficiency gradually increased to 90.6% at 0.06 mg nZVI dosage. Meanwhile, the CuONP efficiency in levofloxacin adsorption increased from 71 (0.01 mg CuONP dosage) to 84% (0.06 mg CuONP dosage). The initial adsorption capacity increase was caused by the bigger surface area and more adsorption sites introduced by increasing the number of adsorbent particles, which also caused the increase of more metals attached to the adsorbent weight (Bayramoglu et al. 2020; Bazrafshan et al. 2019). The decline in the adsorption percentage with the increasing adsorbent dosage for both nano-adsorbents may have been caused by the aggregation of a high adsorbent dose, which reduced the adsorbent surface area. It may also have been caused by the inadequate amount of levofloxacin ions in the solution compared to the available binding sites or by the interference between the higher adsorbent dose and binding sites (Mohammed et al. 2019). Bayramoglu et al. (2020) reported that the adsorbed amount of the dyes decreased from 178.0 to 30.4 mg/g for DR-R and from 471 mg/g to 99.4 mg/g for DV-28 dye by increasing the resin dose from 0.1 to 2.0 g/L. Ogunyemi (2019) described that a high dose of nanoparticles leads to aggregation of nanoparticles and a significant number of adsorption binding sites, which may result in high removal efficiency. Results of the current study also confirmed that CuONPs and nZVI had more binding sites for levofloxacin removal.

#### Effect of contact time on levofloxacin adsorption

The most significant design factor that influencing the efficiency of adsorption processes is the contact time between adsorbate and the adsorbent. The effects of contact time on the CuONPs performance in levofloxacin adsorption were individually investigated. In the CuONPs dosage (0.01) and solution pH (7), we had 50 mL solution volume, 250 rpm agitation speed, and 25 °C temperature. The initial levofloxacin concentrations for all test solutions were 1 mg/L. Figure 5c shows the levofloxacin removal efficiencies as a function of contact times ranging between 15 and 120 min. The data indicate that adsorption immediately started upon the addition of the n ZVI and CuONPs powders to levofloxacin solution. The efficiency of CuONPs levofloxacin removal increased from 27% in 15 min to 71% in 120 min when the equilibrium condition was attained. The percentage of nZVI, levofloxacin removal in the 15 min was 21%. Subsequently, 80% removal was attained when the contact time was continued to 120 min. Large numbers of vacant surface sites were available during the initial stages of adsorption. However, they almost became saturated with levofloxacin and were difficult to occupy as time passed due to the repulsive forces between the bulk phases and the solute molecules on the solid (Githinji et al. 2011). Consequently, the adsorption rate process slowed down during the later phase. Genç and Dogan (2015) suggested that the gradual increase in ciprofloxacin uptake was rapid in the first 30 min and that after 60 min, the adsorption amount remained approximately constant. Our results were consistent with those of previous studies on dye removal (Bayramoglu and Arica 2021).

#### Effect of stirring speed on levofloxacin adsorption

Figure 5D depicts the equilibrium adsorption capacity for different agitation speeds. Higher removal efficiencies were obtained with the stirring speed increase. The percentage removal value increased from 10 to 88% and 22% to 92% for the CuONPs and the nZVI, respectively, when the stirring speed was increased from 100 to 250 rpm, this behavior may be explained by the fact that when the stirring speed increases, all particles are kept in suspension in solution, which enhances the chance of adsorbate contact with the adsorbent (Ghanem et al. 2020). Genç and Dogan (2015) detected that the qe value of ciprofloxacin increased from 84.64 to 98.52 mg g^?1^ by increasing the agitation speed from 100 to 150 rpm. The 250 rpm agitation speed was determined as the optimum speed for the adsorption (Zhu et al. 2018).

#### Effect of temperature on levofloxacin adsorption

Levofloxacin removal efficiency was examined at five different temperatures (25?30?35?45?55 °C). The adsorption capacity and the percentage of levofloxacin removed decreased as the temperature was increased, indicating an endothermic process. At 25 °C, the levofloxacin removal percentage of the CuONPs was 88%. At 35 °C, it decreased to 73%, and (44%) until the temperature reached to 55 °C. At 25 °C, the levofloxacin removal rate of the nZVI was 70%. It increased to 89% at 35 °C and dropped to (70%) at 45 °C, and to (68%) until the temperature reached 55 °C. The equilibrium capacity of the adsorbent for some specific adsorbents changes as a result of the temperature change (Yi et al. 2016). The adsorption capacity increases with the temperature increase because bond breaking on the adsorbent surface causes the pore to enlargement and leads to the development of new active sites. A temperature increase also increases the rate at which the adsorbate molecules diffuse into the adsorbent pores. Levofloxacin eventually occupies all active sites, causing the adsorption process to slow down above 40 °C (Kannan et al. 2010). Turku et al. (2007) reported that at a lower temperature, both removal efficiency and adsorption capacity increased due to active site expansion, which might increase interaction between them and antibiotics. These properties may have contributed to the high adsorption capacity and removal efficiency at 25, and 30 °C. Mohammed et al. (2019) noted that the removal efficiency of ciprofloxacin by zinc oxide nanoparticles increased up to a certain temperature (30 °C) and decreased after it. Chen et al. (2011) suggested that at higher temperatures, adsorbate uptake decreased because of the higher updraft movement of the molecules of the adsorbate. It reduces the attraction between active sites and antibiotics. As at the end of the reaction, it is being controlled by the rate of adsorption at the core, when the attraction between active sites and antibiotics decreased, there would be very little adsorption at a high temperature. It may due to the lower removal efficiency at a high temperature (35 °C).

#### Influence of the initial concentration of levofloxacin

Figure 5F reveals the effect of the initial levofloxacin concentrations (1, 2, 4, 6 and 8 mg/L) on the adsorption process, while keeping operating factors constant (pH?=?7, temperature?=?25 °C, contact time?=?120 min). The adsorption effectiveness of the adsorbent decreased as the starting concentration of levofloxacin was increased. Compared to the nZVI, the CuONPs adsorbent was less affected by the increase in the initial adsorbate concentration. The CuONPs levofloxacin removal efficiency decreased from 89 to 45% at initial concentrations of 1 ppm and 8 ppm, respectively, whereas nZVI achieved maximum removal efficiencies of 91% and 29% at concentration of 1 and 8 ppm, respectively. This decline may have been caused by the limited number of active sites of the nZVI/CuONPs adsorbent, which become more saturated with increase of the antibiotic concentration. Another possible cause is adsorbent site saturation at high adsorbate concentrations. The adsorption level will be independent of the initial concentration as a result of the low initial number of adsorbent molecules relative to the number of active sites available for the adsorbent at low levofloxacin concentrations. Levofloxacin removal is dependent on initial concentration because access to adsorption sites is restricted at higher adsorbate concentrations. Similarly, Ou et al. (2015) suggested that increasing the concentration of levofloxacin solution from (4?24 mg L^?1^) by using a specific amount of magnetic imprinted polymers decreased its removal efficiency from 99.8% to 85.4%. As a result, as adsorbate concentration increased, adsorption capacity decreased while nanoparticle dosage remained constant.

### Adsorption kinetic

#### Adsorption kinetic studies

**Fig. 6:**
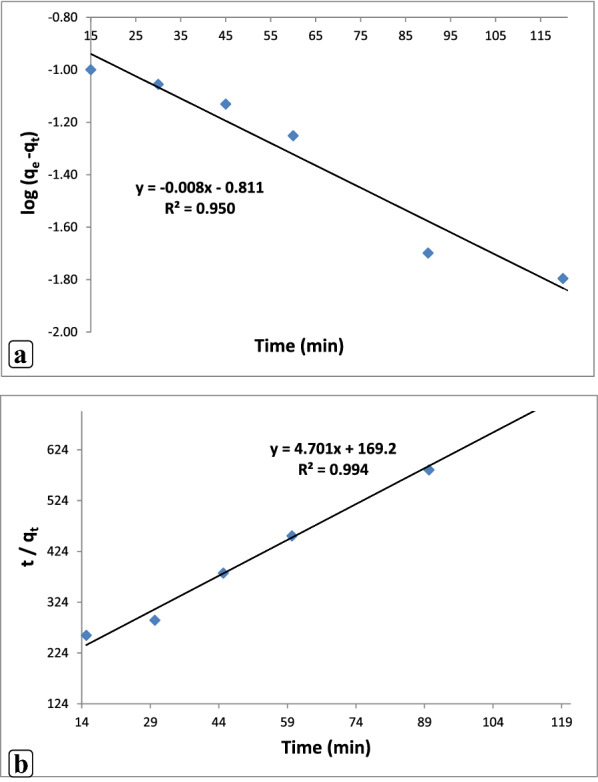
Fitted plots of kinetic models: **a** pseudo-first-order and, **b** pseudo-second-order for levofloxacin adsorption by nZVI

Numerous kinetic models pseudo-primary-order and pseudo-2nd-order were used to explain the kinetic investigations on the adsorptive behaviors of levofloxacin onto the CuONPs and nZVI. Table 2 presents the values of the predicted *q*_*e*_, correlation coefficient (*R*^2^), and rate constant *k*_1_. The plot of *t*/qt against time (*t*) gives a straight line that represents the pseudo-second-order model for levofloxacin adsorption (Fig. 6a and b). Table 2 presents the two parameters equilibrium adsorption capacity *q*_*e*_ and *k*_2_ equilibrium rate constant calculated from the intercept and slope of the curve, respectively. Values of *R*^2^ for the pseudo-second-order model were acquired as 0.983 and 0.994 for CuONPs and nZVI, respectively. These findings support the applicability of the model in describing the adsorptive removal by adsorbent CuONPs and nZVI. The finding revealed that the correlated *R*^2^ values of levofloxacin of the pseudo-second-order model are more significant than those of pseudo-1st-order model, due to the large differences between the values calculated *q*_*e*_ and *Q*e, experimental results indicated that this model to be inadequate for explaining the antibiotic uptake mechanism by the CuONPs and nZVI. In addition, the predicted *q*_*e*_ values of CuONPs (*q*_*e*_, calc 0.159 mg/g) and nZVI (qe, calc 0.98 mg/g) deviated reasonably from their experimental values of qe, exp 0.271 mg/g and 0.19 mg/g, respectively, for pseudo-first-order model. In the pseudo-second-order model, the calculated values of (qe, cal) were found to be in good agreement with the experimental (qe, exp), the predicted qe values of CuONPs (qe, calc 0.123 mg/g) and nZVI (qe, calc 0.12 mg/g). Thus, the kinetic models of CuONPs and nZVI point to the pseudo-second-order, which is commonly applicable by chemisorption of antibiotic onto a homogenous surface. Similar results were reported for levofloxacin removal from aqueous solutions using nano-titanium oxide coated on chitosan (Mahmoud et al. 2020). Altaf et al. (2021) investigated that the levofloxacin adsorption kinetic process in magnetite (Fe3O4) nanoparticles prepared with by green synthesis method, using *Moringa oleifera* plant, and by comparing the pseudo-first order (PFO) and PSO, it was noticed that the pseudo-second-order expression was better adjusted to proceed with the levofloxacin adsorption.

**Table 2:** Kinetic models for adsorption of levofloxacin by CuO NP_S_ and nZVI

Kinetic models	Antibiotic	Parameters		
		CuONPs		
	Levofloxacin	qe,cal (mg g^?1^)	K1 (min^?1^)	R2
Pseudo-first-order		0.159	0.089	0.947
Pseudo-second-order		qe,cal (mg g^?1^)	K2	R2
		0.228	0.06	0.983
		nZVI		
Pseudo-first-order	Levofloxacin	0.98	1.86	0.95
Pseudo-second-order		0.24	0.09	0.994

#### Adsorption isotherm models

**Fig. 7:**
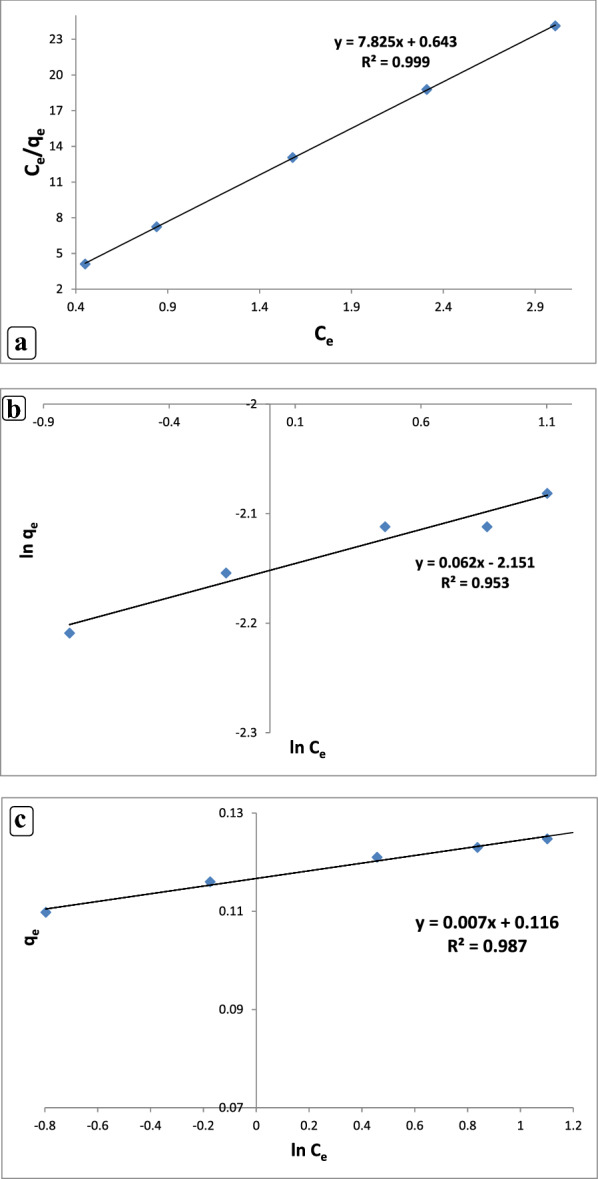
Fitted plots of **a** Langmuir model, **b** Freundlich model, and **c** Temkin model for levofloxacin adsorption by nZVI

Table 3 presents the levofloxacin adsorption isotherm by the CuONPs and the nZVI. The most crucial factor for explaining the relationship between the adsorbent concentration and capacity is the adsorption isotherm. The adsorption behavior of adsorbents during the levofloxacin removal by the CuONPs and the nZVI from the solution was assessed using three common adsorption isotherm models, namely the Langmuir, Freundlich, and Temkin models with varied antibiotic solution concentrations between 1 and 8 mg L^?1^. In the Langmuir isotherm model, solute molecules were distributed in a monolayer pattern on the adsorbent surface. No more adsorption can occur at that location when a solute molecule binds to the active site on the adsorbent. The heterogeneous, multi-layer adsorption of the adsorbate onto the adsorbent forms the basis of the Freundlich isotherm. The Temkin isotherm assumes that the free energy of adsorption and the surface coverage are related. Table 3 shows the outcomes of the Langmuir constant modeling for the CuONP and nZVI adsorbents. The Langmuir isotherm model presented here appeared to have a high *R*^2^ of 0.999 in the adsorption process fitting (Fig. 7a) when *C*_*e*_/*q*_*e*_was plotted against *C*_*e*_. In Table 3, the Langmuir constants (*Q*_*max*_) and separation factors R_L_ and K_L_ were 0.127 and 0.019 (mg g^?1^) and 0.999 (L/mg) and 0.13 and 0.03 (mg g^?1^) and 1.01 (L/mg) for the nZVI and the CuONPs, respectively. R_L_ was less than 1; hence, levofloxacin was suitable under favorable adsorption conditions in the CuONPs and nZVI. The Langmuir isotherm model was found to be the most accurate model for levofloxacin adsorption onto the CuONPs and nZVI, which was quite consistent with the result of Mohammed et al. (2019), who illustrated well-fitting of the Langmuir model for ciprofloxacin adsorption by a zinc oxide-coated pistachio shell with 0.98 correlation. The results demonstrated the homogeneous nature of the biosorbent by the monolayer levofloxacin sorption process on the CuONPs and nZVI. Figure 7b shows the Freundlich isotherm model for levofloxacin adsorption on the CuONP and nZVI adsorbents with *R*^2^?=?0.915 and 0.953, respectively. The adsorption process was slightly fit with the Freundlich isotherm. The constants of the Freundlich isotherm of the CuONPs and the nZVI (i.e., *K*_*f*_ and *n*) in Table 3 were 0.1,6.5,0.11, and 16.1. Levofloxacin adsorbed to the CuONP and nZVI adsorbents at a very considerable extent, as shown by the high amount of *K*_*f*_ constant. Levofloxacin adsorption by the CuONP and nZVI adsorbents also indicated a favorable adsorption system and a multi-layer physical process because *n* was greater than 1. The high value of KF confirms the high adsorption capacity (Boukhelkhal et al. 2016). The high n values obtained from the Freundlich model indicate that the good adsorption of levofloxacin on the CuONPs and nZVI adsorbents is appropriate (Bayramoglu et al. 2020). The result of the present work seems to be in good agreement with those observed by Altaf et al. (2021), who found the data well-fitting of the Langmuir and Freundlich models for levofloxacin adsorption by nano-magnetite with a correlation of 0.954. The fact that the experimental data fit very well with the Langmuir isotherm confirms the monolayer coverage of LEV molecules on CuONPs and nZVI surfaces as well as the uniform distribution of active sites on the surface of the adsorbent (Gebreslassie 2020). The CuONPs and nZVI adsorbents used in this work have a relatively large adsorption capacity (0.12 mg/g and 0.13 mg/g) compared with some other adsorbents reported in the literature, such as aluminum and iron hydrous oxides sorption of ciprofloxacin (0.013 and 0.022 mg/g) (Karthikeyan 2005).This suggests that CuONPs and nZVI are capable of removing Levo from aqueous solutions. According to the Temkin isotherm, the adsorption heat of all molecules decreases in a linear pattern, indicating a homogeneous binding energy. The Temkin isotherm presented in a linear form in Eq. (5) was used to assess the adsorption data. The data analysis revealed that the levofloxacin adsorption data for the CuONP and nZVI adsorbents were well suited with the Temkin isotherm. Fig. 7c displays the linear isotherm constants and *R*^2^. The following values were hypothesized using the Temkin isotherm presented in Table3: AT?=?1.062 L/g and B?=?0.116 J/mol for the nZVI and AT?=?1.24 L/g and B?=?0.101 J/mol for the CuONPs. These values indicate adsorption heat with a physical adsorption process. A graph was created by plotting the quantity sorbed *q*_*e*_ versus *lnC*_*e*_. The correlation coefficient values for the CuONPs and then ZVI in the Temkin isotherm model were 0.955 and 0.987, respectively, which were higher than those in the Freundlich isotherm model, but lower than those in the Langmuir model (Table 3). As mentioned, according to the Temkin isotherm, the adsorption heat of all molecules linearly decreases, demonstrating a homogeneous binding energy (N et al. 2021). The adsorption process was physical for the Temkin isotherm (*R*^2^?=?0.9664) because AT was 0.0723 kJ mol^?1^, which was less than 8 kJ mol^?1^. The results in this study were in agreement with the previous report by Husein et al. (2019), who found that the Temkin isotherm model was applicable in their investigation of the adsorption kinetic process of the drugs molecules (NSAIDs) in Cu NPs. They discovered that the removal of Ibuprofen (Ibu), naproxen (Nab), and diclofenac (Dic) by Cu NPs occurred through a physisorption process.

**Table 3:** Adsorption isotherm parameters for adsorption of levofloxacin by CuO NPs and nZVI

Isotherm models	Antibiotic	Parameters (nZVI)			
Langmuir Freundlich	Levofloxacin	*Q*max (exp) (mg g^?1^)	KL	R2	RL
		0.125		0.999	0.01
		Qmax(cal) (mg g^?1^)			
		0.127			
		KF (mg g^?1^(L mg)1/n)	n	R	
		1.06	0.46	0.953	
Temkin		B	A_T_	R	
		0.116	1.06	0.987	
Isotherm models	Antibiotic	Parameters(CuO NPS)			
	Levofloxacin	Qmax (exp) (mg g^?1^)	KL	R2	RL
Langmuir		0.127		0.993	0.03
Freundlich		Qmax (cal) (mg g^?1^)			
		0.13			
		KF (mg g^?1^(L mg)1/n)	n	R	
		0.109	6.5	0.91	
Temkin		B	A_T_	R	
		0.101	1.24	0.955

#### Influence of temperature and thermodynamic studies

**Fig. 8:**
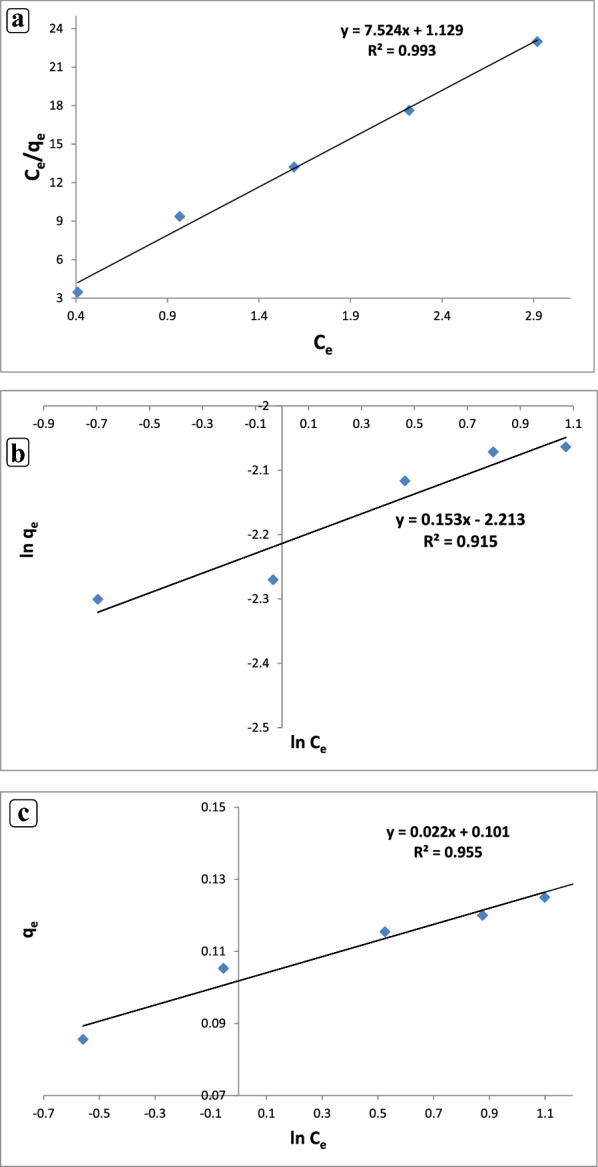
Fitted plots of **a** Langmuir model, **b** Freundlich model, and **c** Temkin model for levofloxacin adsorption by CuONPs

The influence of temperature on the levofloxacin removal by the CuONPs and the nZVI was demonstrated over the 25?55 °C range under optimized conditions of 120 min shaking time, 0.01 g adsorbent, and pH 7 (Fig. 8a and b). Table 4 presents the *G*° values computed using Eq. (10), along with the additional parameters of ?*H*° and ?*S*°. The Van't Hoff equation Eq. (10) and slope and intercept were used to determine the ($\Delta$*H*°) and ($\Delta$*S*°) values, respectively Eq. (11):

**Table 4:** Standard thermodynamic parameters for the adsorption of levofloxacin onto CuONP_S_ and nZVI at different temperatures

Nanomaterials	Antibiotic	Adsorption thermodynamic parameters					
		Temp, *T* (K)	q_e(Exp.)_	?*G*^o^?=? (KJ mol^?1^)	?*H*^o^?=? (KJ mol^?1^)	?*S*^o^?=? (J mol^?1^ K-1)	*R*^2^
CuONP_S_	Levofloxacin	298	0.162	??2.97990388	??28.2759	??0.08488	0.993
		308	0.1482	??2.13104448			
		318	0.1288	??1.28218508			
		328	0.1168	??0.43332568			
nZVI		298	0.1628	??1.80915966	??16.927304	??0.050732	0.999
		308	0.1558	??1.30183938			
		318	0.1486	??0.7945191			
		328	0.14	??0.28719882			

10 $$\Delta G^\circ = \Delta H^\circ -T\Delta S^\circ ,$$

11 $$\mathit{ln}{K}_{c}=\boldsymbol{ }\frac{{-\Delta H}^{^\circ }}{RT}+ \frac{{\Delta S}^{^\circ }}{R}.$$

The plot of *lnK*_*c*_ against 1/*T* refers to a straight line with a slope (???*H*°/*R*) and an intercept (?*S*°/*R*) of the Van?t Hoff (Table 4). The ?*G*° values were between???2.9 and ??0.43 kJ/mol for the CuONPs and between ??1.8 and ??0.28 kJ/mol for the nZVI. The ?G negative free energy change values indicated that both adsorption processes were spontaneous. The ?*H*° values were ??28.2 and ??16.9 kJ/mol for the CuONPs and the nZVI, respectively. The negative charge of the enthalpy change (DH) indicated that the adsorption reactions of levofloxacin antibiotic and the CuONPs and the nZVI were exothermic, indicating a decrease of the temperature-increasing adsorption potential. The CuONPs and the nZVI showed negative Gibbs free energy (?*G*°) values within the range of ??8.0?0 kJ mol^?1^, demonstrating a spontaneous adsorption process and the predominant mechanism of the physisorption process. The calculated ?*S*° values for the CuONPs and the nZVI were???0.08 and???0.05 Jmol^?1^, respectively. The CuONPs and the nZVI showed a negative ?*S*° value of levofloxacin adsorption. This illustrates that randomness decreases on the surface when levofloxacin molecules adsorb on the adsorbent (Al-Kadhi 2020). Kerkez-Kuyumcu et al. (2016) have investigated the ?*H*° value of amoxicillin adsorption on M-GNPs was calculated as ??9.34 kJ mol^?1^, which indicated the adsorption process was exothermic in nature.

### pH at point zero charges (pH PZC) and adsorption mechanism

Results from the pHPZC experiments with CuONPs and nZVI adsorbent, in which the pH ranged from 3 to 12. The pHpzc values of CuONPs and nZVI were 5.4 and 6.1, respectively (supplement materials), indicating that below the pHPZC value, the surface of CuONPs and nZVI were positively charged due to protonation (Balarak et al. 2021), thereby favoring the adsorption of anions. CuONPs and nZVI surface have a greater negative charge than pHPZC, which facilitates the adsorption of levofloxacin. In this instance, the batch adsorption studies used a pH of 7. Since the pHpzc values are 9.5 and 6.1, the positively charged surfaces of CuONPs and nZVI are favored for the adsorption of levofloxacin. Since the pHpzc are 9.5 and 6.1 above, where the surfaces of CuONPs and nZVI are positively charged, hence favors the adsorption of levofloxacin.

### Experimental design

**Fig. 9:**
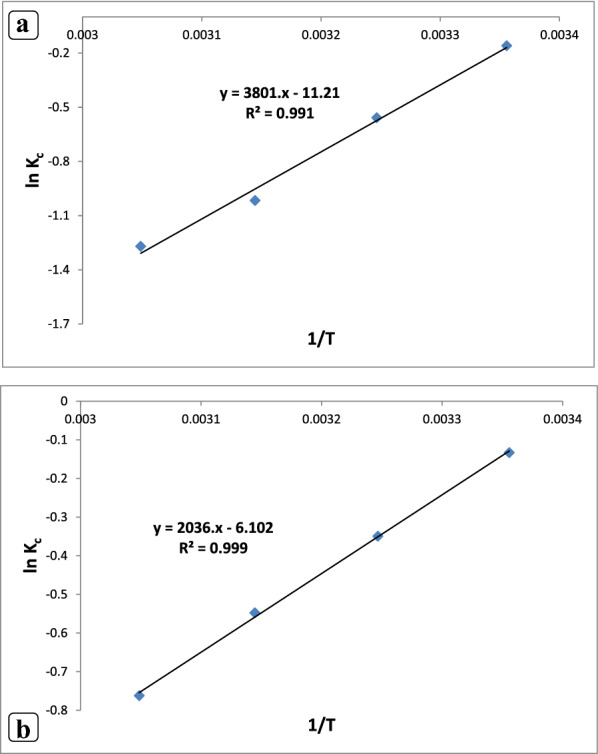
Thermodynamic analysis of levofloxacin adsorption on **a** nZVI and **b** CuONPs

**Fig. 10:**
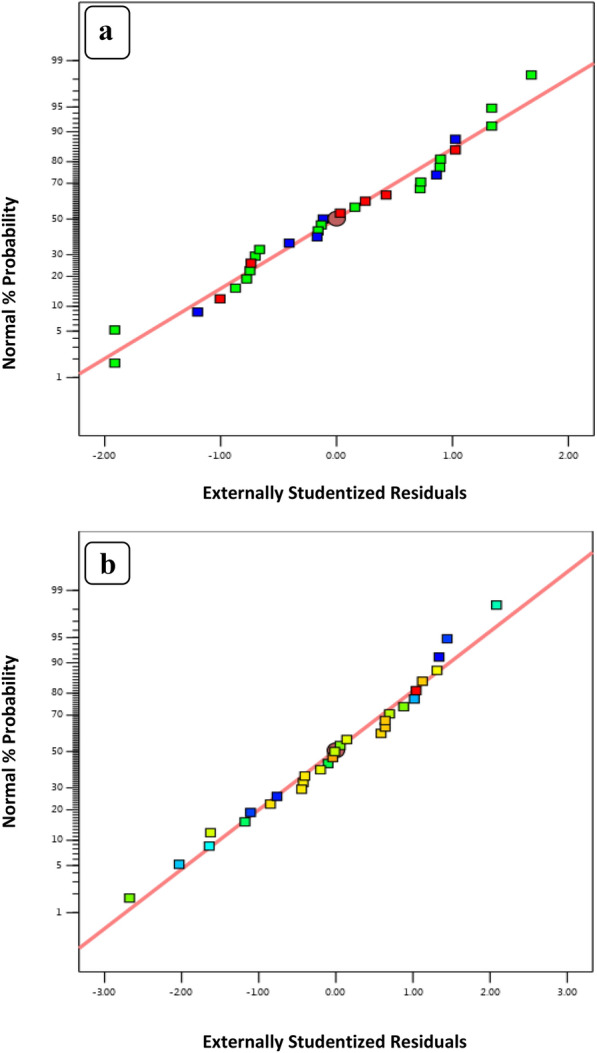
The studentized residual and normal % probability plot of removal of levofloxacin by **a** CuONPs and **b** nZVI

Levofloxacin was removed from the aqueous solution by nZVI and CuONPs, and the Box?Behnken design was used to determine the optimal conditions for this process. Box?Behnken design for four variables (initial levofloxacin concentration, pH, contact time, and adsorbent dosage) with each variable having three levels denoted by the codes minimum (??1), 0, and maximum (1). As input variables, (X1) initial levofloxacin concentration (1?8 mg/L), (X2) pH (3?9), (X3) contact time (15?120), and (X4) adsorbent dosage (0.01?0.06 g/L) were studied. R% is the percentage of levofloxacin that was eliminated as the system?s response. Equation 12 expresses the quadratic equation model for predicting the optimal point:

12 $${\text{Y }} = {\text{B}}_{0} + \, \sum {\text{ki}} = {1}\beta {\text{ixi }} + \, \sum {\text{ki}} = {1 } + \, \beta {\text{iixi2 }} + \, \sum {\text{ki}} = 0\beta {\text{xixj }} + \, \varepsilon ,$$

where *Y* represents the predicted system response and Xi and XJ represent the independent variables of action. The factor ?0 is the model constant; ?i is the linear coefficient; ?ii is the quadratic coefficient, and ?ij is the cross-product coefficient. The quality of the response surface models was expressed by calculating the determination coefficient (*R*^2^) and the adjusted determination coefficient (Adj-*R*^2^). The statistical significance was confirmed by a sufficient ratio of precision and the significance of the F-test. The program Design expert (version 13) was used for regression, contour plot, and graphical analysis. In order to analyze the impact of independent factors on the response, a total of 29 batch runs based on BBD-designed trials with five replicates at the central point were examined. In Eqs. (13 and 14), *Y* is the removal efficiency percent; X1 (dose (g/L), X2 (pH), X3 (contact time (s)), and X4 (initial levofloxacin concentration (mg/L)) correspond to independent variables of CuONPs and nZVI, respectively. The effect of process parameters (independent variables) on levofloxacin adsorption was investigated. Design-Expert 13 was used to compute the coefficients of second-order polynomials for each element of the Eqs. (13, 14). Table 5 lists the experimental and predicted percentage adsorption values (9, 10). The maximum levofloxacin removal efficiency by CuONPs and nZVI particles were 99.4% and 99.8%, respectively. The developed model?s fit was evaluated based on the *R*^2^ and CV values. *R*^2^ values of 0.98 and 0.992 for CuONPs and nZVI, respectively, are relatively high (close to unity). These results indicated that more than 98% of the variations in the removal efficiency of levofloxacin by CuONPs and nZVI were accounted for by the independent factors and that only?>?0.2% of the total variability in the response was not explained by the model (Khosravi and Arabi 2016). The high *R*^2^ value suggested that the approximation of a response in the investigated range may be adequate. The ANOVA results presented in Table 6(a) for this model indicate that the model was appropriate for predicting the adsorption of levofloxacin with a P value less than 0.05. In Table 6(a) and (b), the Model F**-**values of 134.8 and 49.97 for levofloxacin adsorption by CuONPs and nZVI, respectively, indicate that the model is significant. This F-value could only occur due to noise with a probability of 0.01%. When the P-value is less than 0.05, model terms are considered significant. A, B, C, D, A^2^, B^2^, C^2^, D^2^ for CuONPs and A, B, C, D, AB, AD, BD, A^2^, B2, C^2^, D^2^ for nZVI were determined to be significant model terms for the adsorption of levofloxacin by CuONPs and nZVI, respectively. For CuONPs and nZVI removal, the "adequate precision" ratio of the model was determined to be 24.7 and 39.9, respectively. Ratios greater than four indicate that the model has sufficient signal (Manivasagan and Chinnappan 2013). The coefficient of variance (CV) is the ratio of the standard error of estimate to the mean value of the observed responses; it defines the repeatability of the model. Low deviations between observed and predicted values indicate lower CV values for the models. The CuONPs and nZVI removal model CV values were 3.09 and 1.48% (<?10%), respectively, indicating the accuracy and dependability of the experiments (Mekkawi et al. 2021). The CuONPs and nZVIF-values of 0.31 and 2.05 indicated that the established model of removing the efficiency of levofloxacin was insignificant. The non-significant lack of fit suggests the model?s predictability. CuONPs and nZVI had a 94.17 and 25.53% chance, respectively, of having an F-value that was unsuitable due to noise. Figure 9a, b demonstrates that the experimental values were in significant agreement with the predicted values, indicating that the model was satisfactory and precise. Figure 10a and b depicts the normal probability and studentized residual plot for levofloxacin adsorption by CuONPs and nZVI, respectively. Residuals indicate the difference between predicted and actual values, thereby validating the analysis of variance model.

**Table 5:** Box?Behnken design-based experimental conditions for the adsorption of levofloxacin using nZVI and CuONPs

Run	Factor A	Factor:B	Factor:C	Factor: D	Response Factor 1 CuONPs	Predicted value	Response Factor 2 nZVI	Predicted value
1	0	1	1	0	68.15	66.03	74.77	74.78
2	0	0	0	0	70.25	76.05	78.05	77.44
3	0	0	0	0	70.75	76.05	77.05	77.44
4	??1	0	0	1	47.17	55.46	55.15	55.67
5	??1	1	0	0	50.24	46.03	56.12	55.18
6	??1	??1	0	0	52.75	56.97	59.47	58.78
7	0	0	??1	1	71	74.36	77.23	77.81
8	1	0	??1	0	61.5	74.44	72.67	72.07
9	0	??1	0	1	72.5	79.40	77.53	77.12
10	0	1	0	??1	53.75	54.35	61.84	62.89
11	1	0	0	1	63.75	75.11	75.85	75.99
12	0	0	1	??1	65.17	65.36	74.47	73.99
13	1	0	0	??1	52.01	64.07	63.81	62.55
14	0	0	0	0	68.25	76.05	77.03	77.44
15	0	0	0	0	68.25	76.05	77.01	77.44
16	??1	0	0	??1	45.5	44.66	54.35	53.47
17	??1	0	??1	0	52	55.03	56.18	56.92
18	??1	0	1	0	54.25	56.82	60.14	61.38
19	1	0	1	0	66.25	76.47	75.72	75.62
20	0	1	0	1	70.25	66.02	76.25	75.39
21	1	??1	0	0	70.43	79.69	75.25	76.29
22	0	??1	??1	0	75	78.25	77.94	77.19
23	0	??1	1	0	80.25	82.12	83.77	83.07
24	0	0	??1	??1	61.75	67.07	67.14	67.21
25	0	0	1	1	71.25	79.90	79.01	79.04
26	0	0	0	0	68	76.05	78.05	77.44
27	0	??1	0	??1	71	69.24	72.47	73.98
28	0	1	??1	0	67	66.07	72.69	72.65
29	1	1	0	0	55	62.37	66.28	67.07

**Table 6:** Analysis of variance (ANOVA) for the response surface quadratic model for removal of the levofloxacin by (a) nZVI and (b) CuONPs

Source(a)	Sum of squares	*df*	Mean square	F-value	*p*-value	
Model	2067.52	14	147.68	134.89	?<?0.0001	Significant
A-ph	647.83	1	647.83	591.72	?<?0.0001	
B-con	123.39	1	123.39	112.71	?<?0.0001	
C-dose	48.12	1	48.12	43.95	?<?0.0001	
D-time	183.61	1	183.61	167.71	?<?0.0001	
AB	7.90	1	7.90	7.21	0.0178	
AC	0.2070	1	0.2070	0.1891	0.6703	
AD	31.58	1	31.58	28.85	?<?0.0001	
BC	3.52	1	3.52	3.21	0.0948	
BD	21.86	1	21.86	19.96	0.0005	
CD	7.70	1	7.70	7.03	0.0190	
A^2^	897.99	1	897.99	820.22	?<?0.0001	
B^2^	11.69	1	11.69	10.68	0.0056	
C^2^	4.43	1	4.43	4.05	0.0639	
D^**2**^	91.33	1	91.33	83.42	?<?0.0001	
Residual	15.33	14	1.09			
Lack of fit	14.08	10	1.41	4.51	0.0799	Not significant
Pure error	1.25	4	0.3123			
Cor total	2082.85	28				
Std. Dev	1.05			*R*^2^		0.9926
Mean	70.80			Adjusted *R*^2^		0.9853
C.V. %	1.48			Predicted *R*^2^		0.9601
				Adeq precision		39.3323

Source (b)	Sum of squares	*df*	Mean square	*F*-value	*p*-value	
Model	3094.40	14	221.03	49.97	?<?0.0001	Significant
A-ph	1144.26	1	1144.26	258.67	?<?0.0001	
B-con	599.25	1	599.25	135.47	?<?0.0001	
C-dose	10.98	1	10.98	2.48	0.1374	
D-time	357.52	1	357.52	80.82	?<?0.0001	
AB	10.18	1	10.18	2.30	0.1516	
AC	0.0156	1	0.0156	0.0035	0.9534	
AD	0.0156	1	0.0156	0.0035	0.9534	
BC	3.84	1	3.84	0.8684	0.3672	
BD	0.5625	1	0.5625	0.1272	0.7267	
CD	13.14	1	13.14	2.97	0.1068	
A^2^	799.98	1	799.98	180.84	?<?0.0001	
B^2^	87.74	1	87.74	19.84	0.0005	
C^2^	3.60	1	3.60	0.8129	0.3825	
D^2^	170.07	1	170.07	38.45	?<?0.0001	
Residual	61.93	14	4.42			
Lack of Fit	25.26	10	2.53	0.2755	0.9553	Not significant
Pure Error	36.67	4	9.17			
Cor Total	3156.33	28				
Std. Dev	2.10			*R*^2^		0.9804
Mean	68.12			Adjusted *R*^2^		0.9608
C.V. %	3.09			Predicted *R*^2^		0.9358
				Adeq precision		24.7624

13 $$\begin{aligned} &\text{CuONPs}=-8.59725+18.8813*\text{A}+3.14517*\text{B}+-1.88303*C+0.688265*\text{D}\\&+-0.177222*\text{AB}+0.0138889*\text{AC}+0.000816993*\text{AD}+-0.217778*\text{BC}\\&+0.00490196*\text{BD}+0.0473856*\text{CD}+-1.23394*{{\text{A}}^{2}}+-0.408657*{{\text{B}}^{2}}\\&+0.330926*{{\text{C}}^{2}}+-0.00787454*{{\text{D}}^{2}} \end{aligned}$$

14 $$\begin{aligned} &\text{nZVI}=-77.438+7.3475*\text{A}+-3.20667*\text{B}+2.0025*\text{C}+3.91167*\text{D}+-1.405*\text{AB}\\&+-0.2275*\text{AC}+2.81*\text{AD}+-0.9375*\text{BC}+2.3375*\text{BD}+-1.3875*\text{CD}\\&+-11.7661*{{\text{A}}^{2}}+-1.34233*{{\text{B}}^{2}}+0.826417*{{\text{C}}^{2}}+-3.75233*{{\text{D}}^{2}} \end{aligned}$$

#### Effects of variable interaction on levofloxacin removal

**Fig. 11:**
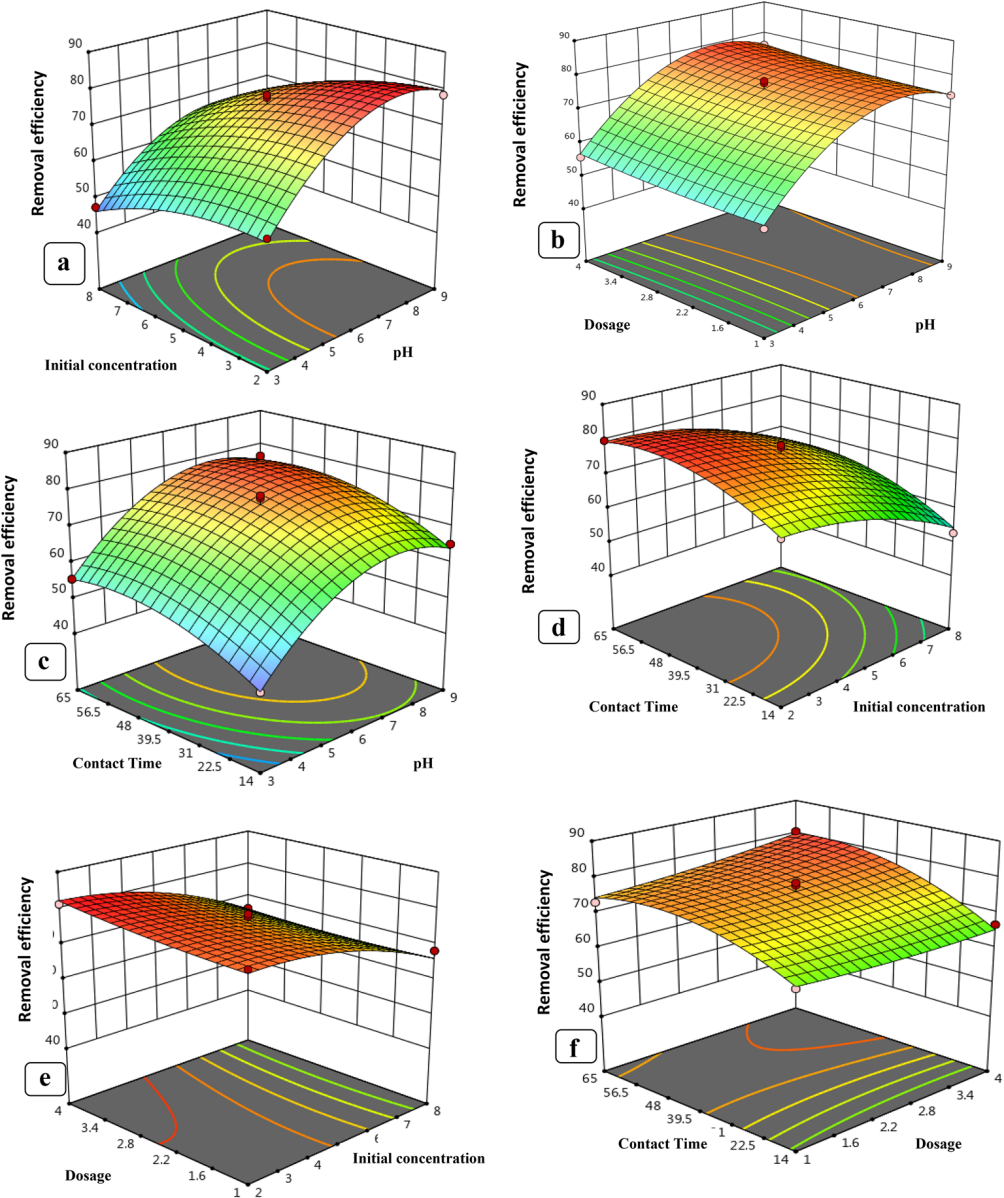

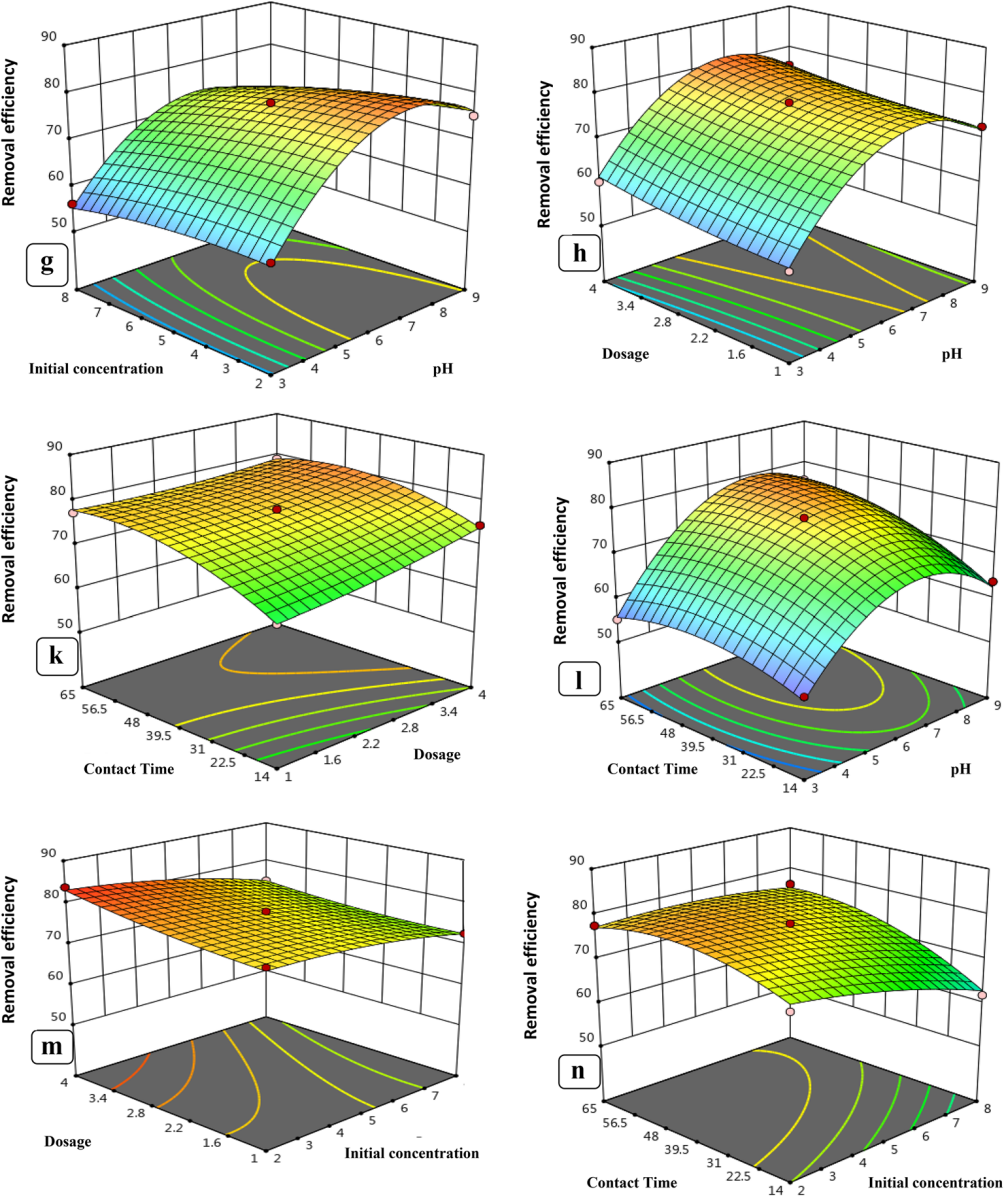
Three-dimensional surface plots depicting interactions among different process variables: **a** initial levofloxacin concentration, pH; **b** adsorbent dose, pH; **c** contact time, pH; **d** contact time, initial concentration; **e** adsorbent dose, initial levofloxacin concentration; **f** contact time, adsorbent dose for CuONPs; **g** initial levofloxacin concentration, pH; **h** adsorbent dose, pH; **k** contact time, adsorbent dose; **l** contact time, pH; **m** adsorbent dose initial levofloxacin concentration; **n** contact time, initial concentration for nZVI

In Fig. 11, the combined effect of nZVI, CuONPs dose, and pH on levofloxacin adsorption at constant initial concentration is depicted. Figure 11a and b demonstrates that pH significantly affects levofloxacin removal; levofloxacin adsorption increased as pH increased. The optimal pH range for levofloxacin adsorption was determined to be between 6 and 7. Figure 11c depicts the combined effect of pH and contact time on levofloxacin removal, which increased as pH and contact time increased within their respective experimental ranges. At pH 7, levofloxacin removal increases with increasing contact time. The pH dependence of levofloxacin adsorption on the adsorbent surface can be explained using the pH and pHPZC of the adsorbent. Due to electrostatic repulsion, a relatively low number of negatively charged sites on the adsorbent surface does not favor levofloxacin adsorption at pH?<?pHPZC, whereas a relatively higher number of negatively charged sites enhances levofloxacin sorption at pH?>?pHPZC (Singh and Bhateria 2020). Wu et al. (2010) reported that at pH 7, levofloxacin existed as a zwitterion/neutral form, which made its dissociation and interaction with the adsorbent iron oxide nanoparticles easier. Altaf et al. (2021) reported that a maximum removal efficiency of 86% for levofloxacin was achieved by chemically synthesized magnetite at pH 6.5. Altaf et al. (2021) reported that a maximum removal efficiency of 86% for levofloxacin was achieved by chemically synthesized magnetite at pH 6.5. The interactive effect of initial levofloxacin concentration and contact time on levofloxacin removal is represented in Fig. 10e. In low levofloxacin concentrations, the ratio of surface active sites to total molecules is high, so all molecules stick to the nZVI and CuONPs surfaces and are then removed from the solution. As the initial concentration of levofloxacin increased, the efficiency of removal first increased and then decreased. However, there are not enough spaces for all molecules in high concentration of levofloxacin. Al-Jabari et al. (2019) used chemically synthesized magnetite and reported a maximum removal efficiency of 82.2% with 100 mg L^?1^ nanoparticles for 2.5 mg L^?1^ levofloxacin concentration. Similarly, Fig. 11f depicts the combined effect of nZVI, CuONPs dosage, and initial levofloxacin concentration on levofloxacin removal. Levofloxacin removal increased as nZVI increased, the CuONPs dose increased from 0.01 mg/L to 0.06 mg/L, and the levofloxacin concentration decreased. Because the adsorption sites and surface area of the nZVI and CuONPs get saturated when the initial levofloxacin concentration increases, the adsorption effectiveness decreases (Zhou et al. 2018). The maximal levofloxacin removal was determined to be 79.9% and 82.7%, respectively, at nZVI and CuONPs doses of 0.03 g/L and levofloxacin concentrations of 2 mg/L.

### Process optimization for levofloxacin adsorption

Multiple response optimization was used to optimize any combination of the five objectives: initial levofloxacin concentration, initial solution pH, nanomaterial concentration and contact time, and levofloxacin removal efficiency. The purpose of the adsorption was to define the sorption process for an initial levofloxacin concentration of 1?8 mg/L, an nZVI, and CuONPs dose of 0.01?0.03 mg, an adsorption contact time of 10?120 min, and an initial solution pH of 3.0 to 9.0. At an initial solution pH6.0, 45 min of contact time for nZVI and CuONPs, adsorbent dosage of 0.03 g/L, and an initial levofloxacin concentration of 5 mg/L, the maximum removal efficiencies of levofloxacin were obtained, and at these optimal conditions, the predicted levofloxacin removal efficiencies were 78.2% and 79.9% with desirability values of 1.000 for nZVI and CuONPs, respectively.

### RP-HPLC analysis method

**Fig. 12:**
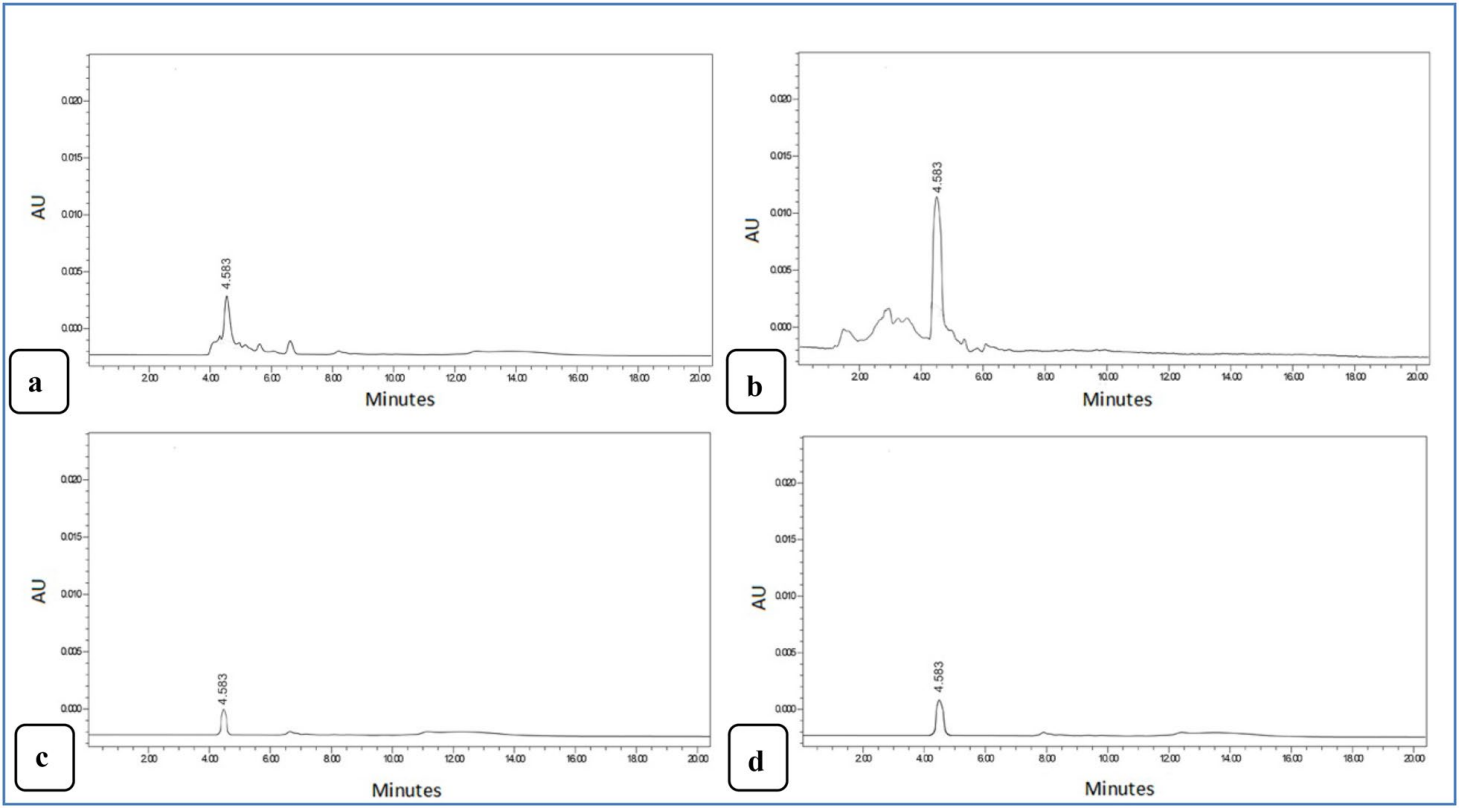
HPLC chromatogram of levofloxacin was1.02 mg/mL in drain (**a**) levofloxacin was 4.7 mg/L of wastewater of hospital (**b**), after treatment by using nZVI was 0.3 mg L^?1^ (**c**) and **d** 0.8 mg L^?1^ after using CuONPs

We aimed hereinto determine the antibiotics present in the wastewater of the Bilbeis drain and hospital and analyze the efficiency of the CuONPs and the nZVI in treating influents. The isocratic elution was used at 1 mL min^?1^ flow rate, with UV detection at 294 nm. Figure 12a and d and d represents a chromatogram of the intact LEVO before and after treatment using nZVI of 0.3 and 0.83 mg L^?1^after the CuONP usage. The results showed a great decline in the peak area of LEVO, which was an evidence of its degeneration. No further peak was found, demonstrating that the drug was degraded to H_2_O and CO_2_.

### Effects of nZVI and CuONPs treatment on ARGs variations

**Fig. 13:**
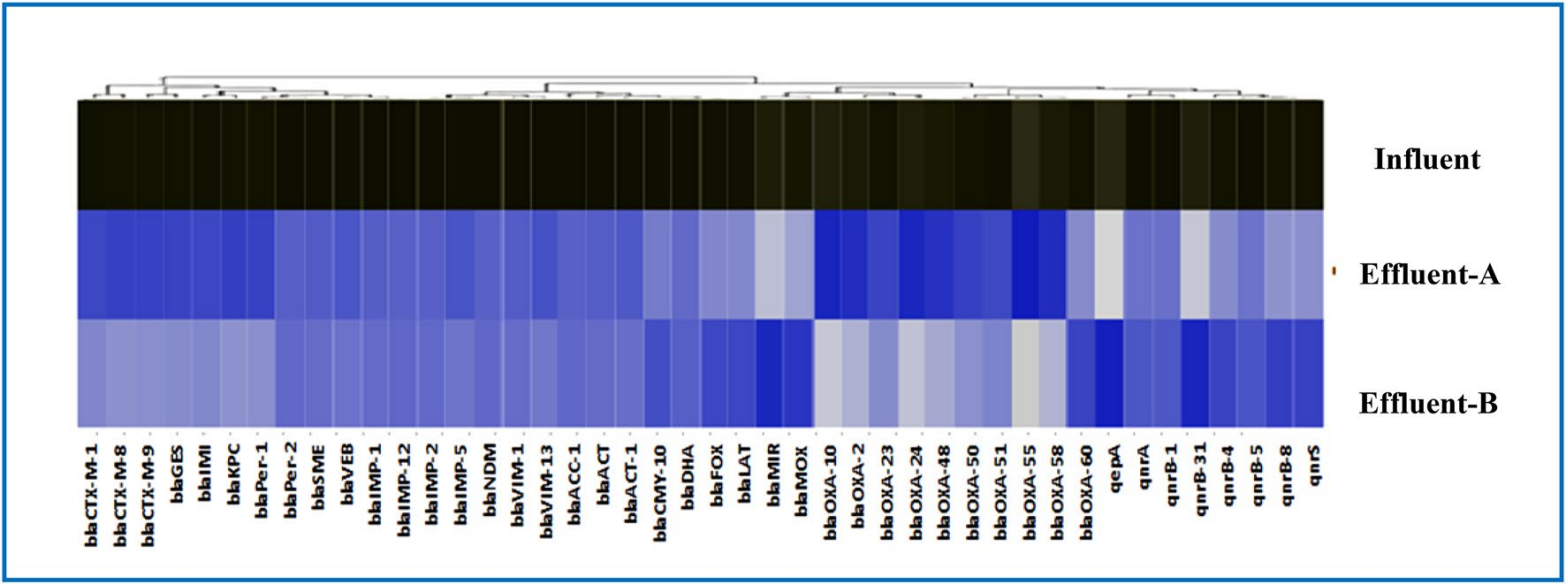
Heatmap analysis of the relative abundance of antibiotic resistance genes present in the influent, effluent-A and effluent-B

The removal of ARGs from wastewater by nZVI and CuONPs at optimal conditions of pH 7.22, 1 g/L and 120 min. The hospital?s raw water samples contained 37 ARGs from five distinct classes. The concentrations of ARGs decreased significantly (*t*-test, *p*?<?0.04) from 3.6?×?10^4^copies/mL to 8.2?×?10^6^ and 3.1?×?10^4^ copies/mL to 7.2?×?10^6^were (t-test, *p*?<?0.04) for Class A ?-Lactamase genes and Class B ?-Lactamase genes (bla CTX-M-1,bla CTX-M-8, bla CTX-M-9, bla per-2, blaSME, and blaVEB) and Class C B- (bla 1MP-1, respectively). Class A ?-Lactamase gene frequencies ranged from 1.4% to 95.8%, while Class B ?-Lactamase gene frequencies ranged from 1.4 to 95.8% and 8.3% to 41.1%. The CTX-M-1, bla KPC, blaACC-1, and blaVIM-1 genes had the highest frequency in that order. In contrast, concentrations of the ARGs, which varied from 3.2?×?10^4^ copies/mL to 6.8?×?10^6^ and 4.7?×?10^4^ copies/mL to 7.1?×?10^6^ were reduced significantly (*t*-test, *p*?<?0.05) for Class C ?-Lactamase genes and Class D ?-Lactamase genes (bla (ACC-1, DHA, LAT, MIR, FOX, and bla MOX)) and (bla (OXA-2,OXA-24, OXA-48, and OXA-60), respectively. Frequencies of class C ?-Lactamase genes ranged from 7.2% to 72.3%, while class D ?-Lactamase genes ranged from 7.2%, to 72.3% and 5.2% to 82.1%, respectively. The blaACC-1, blaMIR, bla OXA-55 and bla OXA-10 genes had the highest frequencies. The concentrations of the ARGs varied from 2.9?×?10^4^ copies/mL to 8.3?×?10^6^ were reduced significantly (*t*-test, *p*?<?0.05) for fluoroquinolone resistance genes, qepA (qnrA,B-1,B-5,B-8 and qnrS), respectively. The frequency of fluoroquinolone genes in the category varied between 7.2% and 80.3%, where the qnrB-31 genes were found to have the highest frequencies. Markkanen et al. (2022) discovered the carbapenemase genes blaKPC, blaNDM, blaIMP, blaOXA-48, and blaOXA-58 in hospital wastewater samples from Burkina Faso and Finland. It was believed that *Acinetobacter baumannii*, an opportunistic pathogen is known to cause nosocomial infections, was almost exclusively associated with the blaOXA-58, blaOXA-51, and blaOXA-23ARGgenes (Cacace et al. 2019). Hou and Yang (2015) observed that the bla_CTX-M_ resistance gene was the predominant gene associated with (80.8%) *E*. *coli* (Action et al. 2021). The efficiency of ARG removal by nZVI and CuONPs is depicted in Fig. 13 after 1 g/L nZVI, the removal rates for Class A ?-Lactamase genes and Class B ?-Lactamase genes had the highest removal rates in the present, by 43.5% to 79.8% and 47.2% to 94%, respectively, followed by Class C ?-Lactamase genes and Class D ?-Lactamase genes, by 56% to 77.8% and 54% to 99.2%, respectively, and fluoroquinolone genes had the lowest removal rate, with 34.4% to 89.4%. In contrast, Class A ?-Lactamase genes and Class B ?-Lactamase genes had the highest removal rates, by 30.6%to 75.7% and 43.3% to 77.1%, respectively, followed by Class C ?-Lactamase genes and Class D ?-Lactamase genes, with 39.9% to 84.1% and 33.2% to 81.3%, respectively, and fluoroquinolone genes had the lowest removal rate, with 34.4% to 89.4%. These results suggest that CuONPs treatment had a lower ARG removal efficiency than nZVI. Generally, ?-lactam resistance genes were more prevalent in the raw influent than in the effluent, except blaGES-23 and blaOXA-58, found throughout the treatment plant. Similar to previous findings, five ?-lactam ARGs (blaOXA-1, bla(OXA-10, DHA-1, SHV-1, and TEM-1) and two quinolone ARGs (qnrA and qnrD) cannot be effectively removed by sodium hypochlorite disinfection (Yao et al. 2021). The nZVI is cytotoxic, according to Changha et al. (2008); it can interact with functional proteins on cell membranes, destroying the integrity of microbial cells. The porous passivation layers might be helpful for adsorption under neutral and alkaline conditions (Zhang et al. 2020).The increased deprotonation of iron oxides Fe^0^ layer at higher pH increases the electron availability at reactive surfaces (Rajajayavel and Ghoshal 2015). Zhang et al. (2020) reported that after 120 min of treatment, S-nZVI reduced the amount of 16S rRNA in water from 1.25?×?10^11^ to 2.24?×?10^7^ copies. The effect of sufidated nanoscale zero-valent iron (S-nZVI) with different S/Fe ratios may lead to diverse bacterial dysfunction by influencing the superficial functional groups, status of active sites, electron transfer, structures, and surface area. The shifting surface properties might cause different levels of DNA damage by affecting the cytotoxicity of NPs (Cheng et al. 2019), and further affect the bacterial regrowth. Chen and Zhang (2013) reported that coagulation with FeCl_3_ and PFC at a concentration of 18 mg/L improved the treatment efficacy of several ARGs by 1.15 to 2.46 log. Compared to untreated sewage, reverse osmosis and nanofiltration were 4.98?9.52 logs more effective at removing sulfonamide and tetracycline resistance genes (Lan et al. 2019). After adding 4 mM of nano-iron to tet A, tet C, sul I, sul II, and intI1 in raw water, the concentration of ARGs in water decreased significantly, according to Sun et al. (2021). These respective ARG concentrations decreased from 1.26?×?10^5^copies/mL to 2.40?×?10^4^ copies/mL.

### Microbial community structure in the influent and effluent

**Fig. 14:**
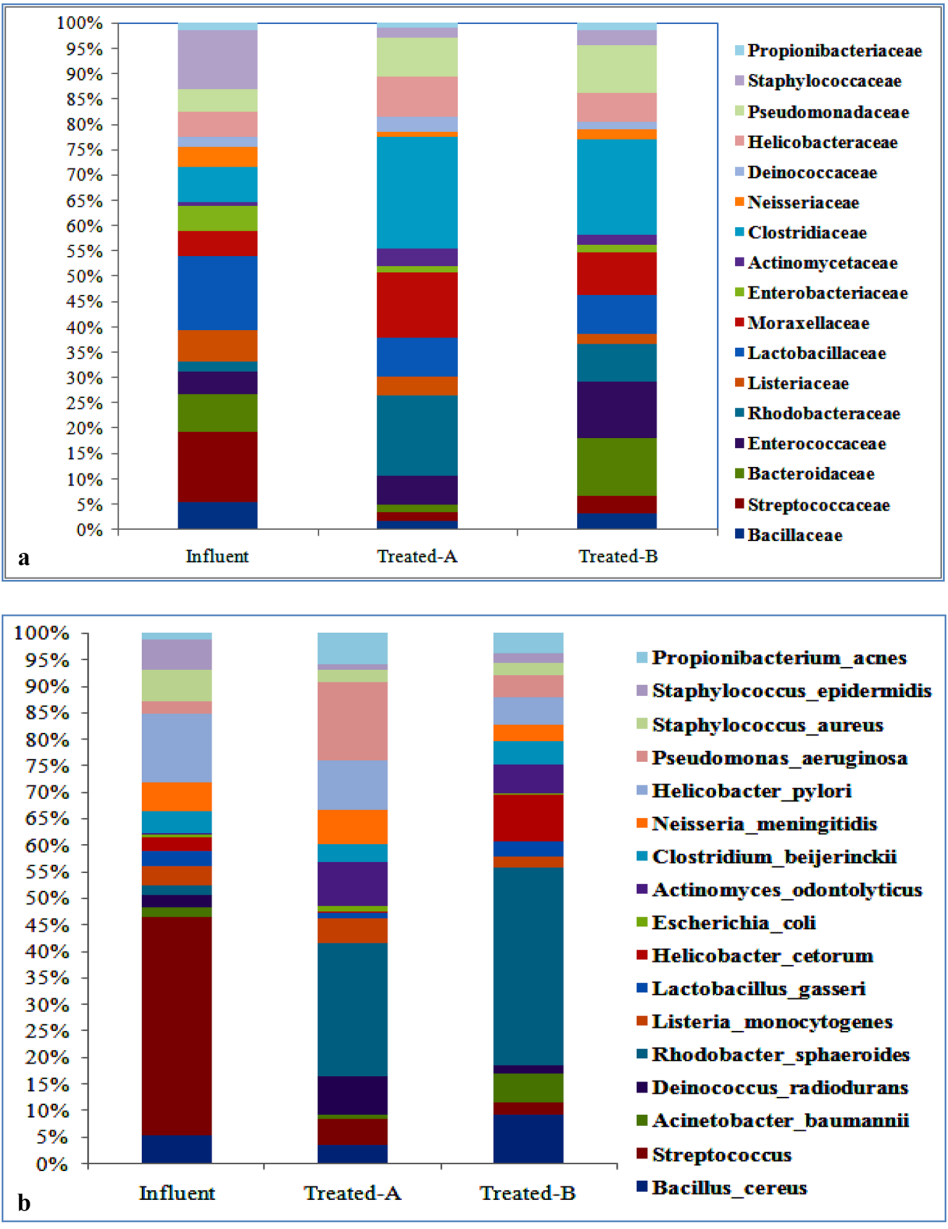
Bacterial composition of the bacterial community at the levels of phylum (**a**), and **b** species of RAW and treated by nZVI and CuONPs water samples

**Fig. 15:**
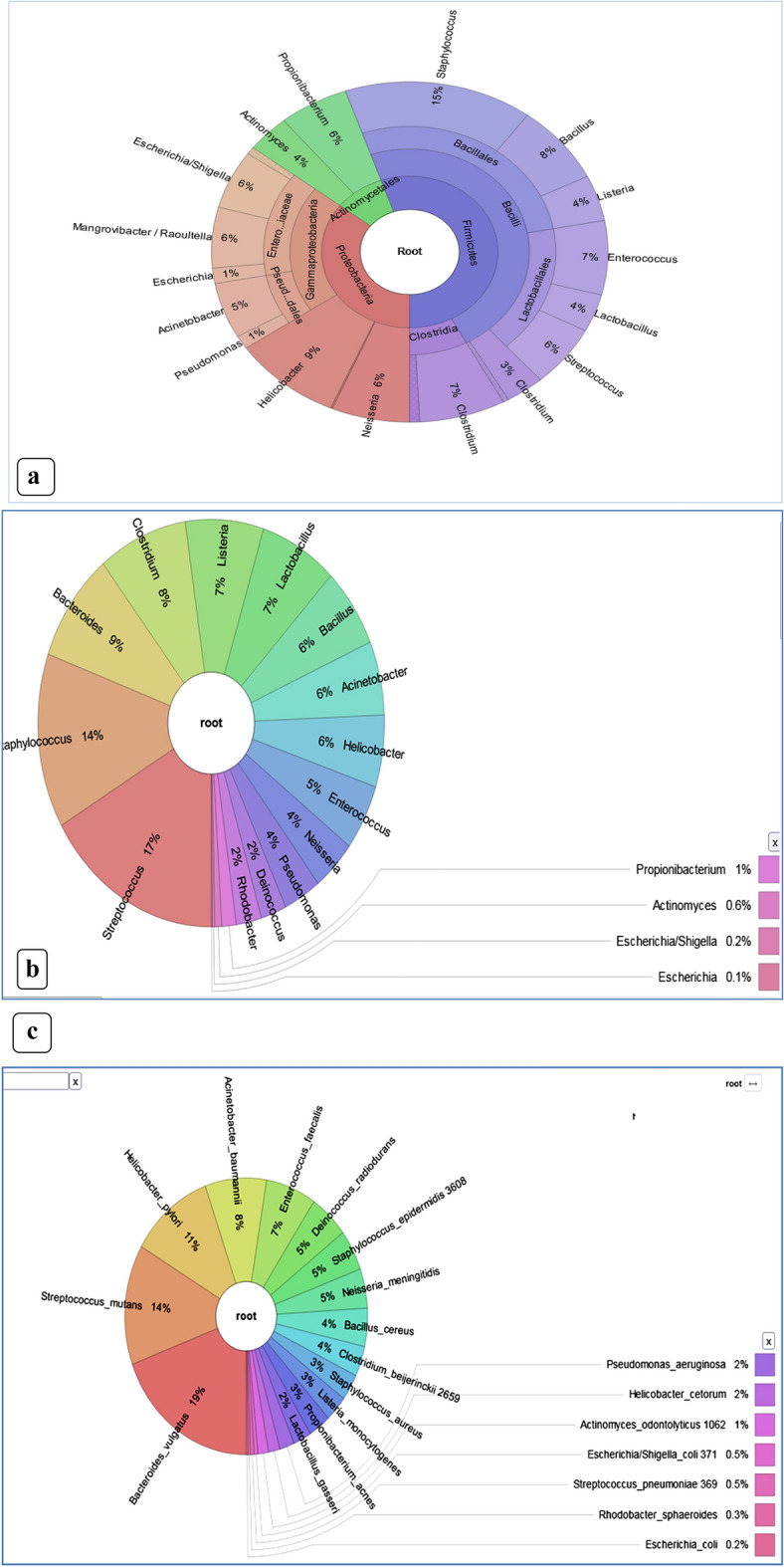
Krona graph showing the relative abundance at the level of **a** phylum, **b** genus and **c** species

By sequencing 16S rDNA, the bacterial community structures of samples from influent to effluent A and B were analyzed, as depicted in Fig. 13a?c. The main bacterial phyla in raw water were *Proteobacteria* (35%), *Firmicutes* (53%), *Bacteroidetes* (7%), and *Deinococcus?Thermus* (5%). At the family and genus levels, the population composition of bacteria in the influent and outflow samples was distinct. Although18 families were identified at???1% relative abundance in influent, microbial communities in the three samples were clearly distinct at the family level. After treatment by nZVI and CuONPs, the percentage of *Streptococcacea*e in treated water which accounted for nearly 18%, decreased to 2.3%?±?0.005 and 4.9%?±?0.052 for the influent sample, respectively. The relative abundance of *Staphylococcaceae* (23178 OTUs)decreased from 13%?±?0.16 in raw water to 2.8%?±?0.011 and 4.7?±?0.021 in water treated with nZVI and CuONP-treated water, respectively. The analysis of the bacterial community at the family level revealed that the relative abundance of members of the class *Firmicutes*, which includes the *Lactobacillaceae*, *Bacillaceae*, and *Clostridiaceae*, contained several human pathogens decreased significantly (*p*?<?0.005) upon treatment, from 29%?±?0.88 in raw water to 1.15%?±?0.06 to 3.5?±?0.08 for nZVI and 5.7?±?0.49 for CuONP-treated samples, respectively. In contrast, members of the class *Gammaproteobacteria*, Gram-negative bacteria from the families *Enterobacteriaceae* (9898 OTUs) and *Pseudomonadales* (9898 OTUs), were analyzed (8413 OTUs). The relative abundance of *Pseudomonadaceae* increased from 5.4% in raw water to 6.4%?±?0.05 and 8.1%?±?0.06 in both effluents. The relative abundance of *Enterobacteriaceae* decreased from 4.1% in raw water to 1.7%?±?0.02 and 2.4%?±?0.06 in both effluents, the relative abundance of *Pseudomonadaceae* increased to 6.4%?±?0.05 and 8.1%?±?0.06 in both effluents compared with 5.4% in raw water. Consistent with our previous findings, we observed that the relative abundance of *Pseudomonadaceae* increased to 4.3% in effluent compared with 1.1% in raw water. The *Neisseriaceae *(6861 OTUs) dominated *Betaproteobacteria* (influent?=?4.1%, effluent?=?1.9%?±?0.05?=?2.2%?±?0.05 for nZVI and CuONPs, respectively). The same fluctuation was detected for members of the classes that contain bacteria frequently present in the human gut, such as *Helicobacteraceae* (9546 OTUs), which prevalence dropped from 5.1%?±?1.08 to 1.66%?±?0.02 and 1.8%?±?0.09 upon wastewater treatment (*p*?<?0.001). The human gut-associated family *Bacteroidaceae* (14687 OTUs) was found in greater abundance in raw water (9.9%) than in effluent (2.09%?±?0.048) and 3.12%?±?0.047%) in nZVI and CuONPs treated water. A fluctuation in the opposite direction was seen for classes that contain mainly environmental, non-pathogenic, bacteria as *Rhodobacteraceae* (3986 OTUs), whose presence after treatment increases from 2.3%?±?0.13 in raw water to 2.95%?±?0.036 and 3.45?±?0.046 in nZVI and CuONPs, exhibited a fluctuation in the opposite direction. In this study, the abundances of *Bacteroidaceae, Rhodobacteraceae and Pseudomonadaceae increased with increasing* nZVI and CuONPs *concentrations.* These bacteria phyla have been reported to be frequently associated with antibiotic resistance (Jiang et al. 2019)*.* Previously, exposure to CuO and ZnO NPs contributed to the enrichment of *Pseudomonas* and *Thiopseudomonas*, dominant genera within the *Proteobacteria* phylum, and their abundances were largely increased by exposure to ZnO and Fe^0^ NPs (Huang et al. 2019). *Propionibacteriaceae* (2242 OTUs), *Deinococcaceae* (3995 OTUs), *Moraxellaceae* (9815 OTUs), and *Listeriaceae* (12179 OTUs) were detected in influent at concentrations ranging from 2 to 5% following treatment by nZVI a, with a decrease in the range of 1.03%?±?0.06 to 2.5%?±?0.08 and 1.9%?±?0.06to 3.6%?±?0.06 for CuONPs, respectively. The community profile variation was observed in the species-level bacterial community analysis. According to Figs. 14 and 15, the top three species with the highest prevalence in the raw water source were *Bacteroides vulgatus, Streptococcus mutans,* and *Helicobacterpylori,* while *Escherichia coli* and *Bacilluscereus* were the top two in the effluent source. Only 17 out of 20 species decreased in concentration from an influent to an effluent source. Epsilonproteo bacteria species*, Helicobacter pylori* (8387OTUs), and *Helicobactercetorum* (1158 OTUs), and one species from the *Betaproteo* bacteria and *Alphaproteo* bacteria classes accounted for 1% of the OTUs. These species were reduced in effluent by 86% to 93.7% for nZVI and by 84.5% to 89.7% for CuONPs. The most numerous species belonged to the *Bacilli* class (six), followed by two species in (*Streptococcus mutans* and *Streptococcus pneumoniae* (369OUTs), *Staphylococcus_aureus* (2333OTUS), and *Staphylococcus epidermidis* (369OUTs), and one species in each of Enterococcus, *Lactobacillus gasseri* (1827OTUs) and *Listeria_monocytogenes* (2286OTUs), respectively. In effluent, these species were reduced by 88.03% to 95.6% for nZVI and by 82.4% to 93.8% for CuONPs. *Clostridium beijerinckii* had 2659 OTUs, *Deinococcus radiodurans* had 3948 OTUs*,* and *Bacteroides vulgatus* had 14575 OTUs*,* accounting for 12%, 7*%,* and 27% of the total, respectively*.* These species were reduced by 91.8% to 95.01% in the effluent for nZVI and by 89.8% to 91.4% for CuONPs. The metagenomic analysis provided us with broad profiles of pathogenic bacterial species in effluent and raw water samples. In this study, we observed relative increases in the diversity of known pathogenic bacteria in hospital wastewater. Additionally, we discovered that nZVI and CuONPs reduce the proportion of Phyla *Proteobacteria* and *Firmicutes* present in water, especially classes such as *Gammaproteo bacteria* and *Bacilli*, which include a wide range of opportunistic pathogens and commensals. In this context, it is essential to note that *Proteobacteria* and *Firmicutes* are among the most prevalent human bacterial pathogens. Based on metagenomics, the pathogens *Enterococcus faecium*, *Staphylococcus aureus*, *Acinetobacter baumannii*, and *Pseudomonas aeruginosa* were frequently detected in both types of samples. Several of these organisms belong to the ESKAPE pathogen group, which has been identified as the bacterial species most likely to develop multiple antibiotic resistance (Lira et al. 2020). These organisms are hubs for the acquisition and spread of ARGs and as such, they should be avoided(Yasir 2021). Tong et al. (2019) discovered that 15 species of potential pathogens, including *Enterobacter*, *Escherichia/Shigella*, *Enterococcus*, and *Streptococcus*, had a direct relationship with ARGs, suggesting that these species may play a role in the spread of ARGs in wastewater. According to the findings of a previous study, *Acinetobacter* is a prevalent emerging opportunistic infection that has been discovered in hospitals. For example, some antibiotic-resistant strains of *Acinetobacter baumannii* are released from hospital wastewater into municipal sewage(Alexander et al. 2020). Antibiotic-resistant *E. coli* strains in hospital wastewater increase the likelihood of ARG transfer to *Enteropathogens* (Wanget al. 2017).Our research demonstrates the significance of monitoring and controlling the incidence of specific pathogens in the effluent, primarily due to the detrimental effects on human health when treated water is reused for various purposes, such as crop irrigation. Furthermore, their distribution through surface water may affect the propagation of particular pathogens and their persistence in the ecosystem. Previous research has shown that monitoring fecal contamination indicators cannot replace the monitoring of specific microbial pathogens.

### Reusability of CuONPs and nZVI

**Fig. 16:**
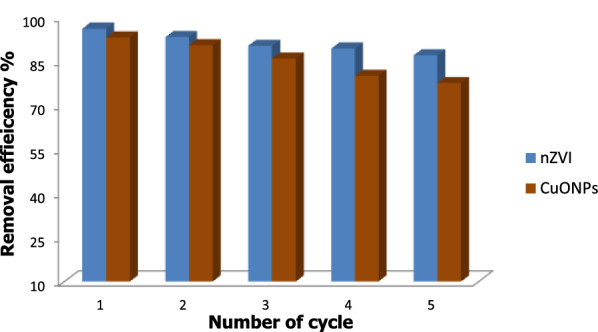
Reusability of nZVI and CuONPs in levofloxacin adsorption

Every adsorbent intended for use in large-scale applications must be recyclable. In this competition, the five-cycle reusability of CuONPs and nZVI were evaluated. CuONPs and nZVI were easily separated by centrifugation after levofloxacin adsorption, washed with ethanol (99%) as the desorption medium, dried in an air oven at 65 °C for 2 h, and then tested for the subsequent adsorption run. In five cycles, levofloxacin elimination ranged from 95.98% to 87.05% and 93.03% to 77.55% (Fig. 16). These results are consistent with those of (Bayramoglu et al. 2020) who reported that the five desorption cycles of terpolymer resin efficiencies were 89.4% and 91.7% for DR-R and DV-28 dyes, respectively.

## Conclusion

Recently, there has been significant interest in innovative treatment methods. Nanotechnology is currently focused on the synthesis of magnetic nanocomposites for the elimination of contaminants, particularly antibiotics. Although antibiotics have been present in the environment for a long time, only in the last few decades, have adverse effects been identified. Although numerous studies have been conducted on their acute and long-term effects on flora, wildlife, and humans, their individual and combined health effects remain unknown. The environmental fate, effect, and potential dangers of these antibiotics require a deeper understanding. This study investigated the removal of antibiotics and ARGs from hospital wastewater treated with nanomaterials nZVI and CuONPs. At an initial solution of pH6.0, 45 min of contact time for nZVI and CuONPs, adsorbent dosage 0.03 g/L, and an initial levofloxacin concentration of 5 mg/L, the maximum removal efficiencies of levofloxacin were obtained, and at these optimal conditions, the predicted levofloxacin removal efficiencies were 78.2% and 79.9% with desirability values of 1.000 for nZVI and CuONPs. The adsorption capacity of the nZVI to LEV was higher than that of the CuONPs. The model simulation results revealed that the adsorption kinetic data were consistent with those of the pseudo-second-order kinetic model. The adsorption equilibrium data of the LEV adsorption onto the nZVI and the CuONPs were best described by the Langmuir model with a *R*^2^?=?0.993 and 0.999 and the Temkin isotherm model with *R*^2^?=?0.987 and 0.955, respectively. The LEV adsorption onto the nZVI and the CuONPs were nonspontaneous and exothermic in nature. Our findings highlight the importance of monitoring and regulating the presence of specific pathogens in the effluent of wastewater treatment plants, primarily due to the detrimental effects reused water has on human health. Additionally, the distribution of certain pathogens in surface water may affect their transmission and persistence in the environment. *Escherichia coli*, *Acinetobacte*r, *Enterococcus*, *Streptococcu*s, and *Pseudomonas*, were among the harmful bacteria found in high concentrations in untreated hospital wastewater, necessitating their removal. Consequently, the use of nano-zero-valent and nano-copper as a tertiary treatment is a promising method for improving the quality of effluents produced for the protection of the environment and human health. The synthesized nZVI and CuONPs are cost effective and eco-friendly adsorbents with high reusability.

## Data Availability

All data generated or analyzed during this study are of our own work and it is our pleasure to be available publically.
